# Spectral Similarity-Based Grouping Improves Laboratory Vis-NIR Prediction of Soil Organic Matter Under Soil Heterogeneity

**DOI:** 10.3390/s26216824

**Published:** 2026-10-28

**Authors:** Jia Deng, Junjie Liu, Huixia Li, Bifeng Hu, Jie Peng, Zijin Bai

**Affiliations:** 1 College of Agriculture, Tarim University, Alar 843300, China; 2 Department of Land Resource Management, School of Public Administration, Jiangxi University of Finance and Economics, Nanchang 330013, China; 3 Philosophy and Social Sciences Laboratory of Data Science in Finance and Economics at the Ministry of Education, Jiangxi University of Finance and Economics, Nanchang 330013, China; 4 State Key Laboratory of Soil Pollution Control and Safety, Zhejiang University, Hangzhou 310058, China; 5 Key Laboratory of Genetic Improvement and Efficient Production for Specialty Crops in Arid Southern Xinjiang of Xinjiang Corps, Alar 843300, China; 6 Key Laboratory of Tarim Oasis Agriculture (Tarim University), Ministry of Education, Alar 843300, China

**Keywords:** autoencoder clustering, Chinese Soil Spectral Library, spectral similarity, local modeling, SOM prediction

## Abstract

**Highlights:**

**What are the main findings?**

- A highly accurate soil prediction method based on hyperspectral similarity is proposed, which outperforms traditional zonation modeling schemes.
- The AEC was the best-performing method in this study for implementing this similarity-based prediction scheme.

**What are the implications of the main findings?**

- In the evaluated dataset, spatial proximity was not consistently associated with spectral similarity.
- Hyperspectral features varied significantly even within the same soil type in this dataset, suggesting that soil type alone was insufficient for spectral-based SOM prediction.

**Abstract:**

Laboratory visible–near-infrared (Vis-NIR) spectroscopy is an efficient and practical approach for measuring soil properties. However, its predictive accuracy is often limited by spectral variability resulting from soil heterogeneity. Such variability poses challenges for traditional global spectral modeling methods in capturing the highly complex mapping relationships between spectral features and soil attributes. To address this issue, this study introduces a novel spectral similarity-based partitioned modeling strategy and compares it with a soil type-based grouping approach. The proposed method clusters soil samples according to their spectral characteristics to minimize within-group variance. Subsequently, individual inversion models are developed for each cluster, enabling more precise learning of stable relationships between specific spectral patterns and soil properties, thereby improving both model accuracy and interpretability. To validate this methodology, the study systematically evaluates the performance of five traditional clustering algorithms (TCAs) and five deep clustering algorithms (DCAs) using the Chinese Soil Spectral Library (CSSL) and the national soil spectral library of China, supplemented with local laboratory Vis-NIR data from Xinjiang, targeting soil organic matter (SOM). Results indicate that both spectral similarity-based and soil type-based grouping strategies significantly surpass conventional global modeling methods. Among them, the spectral similarity-based partitioning approach yielded the most notable improvements. Compared with traditional global modeling, it increased R^2^ from 0.65–0.71 to 0.81–0.85 (relative increase: 14.08–30.77%) and reduced RMSE from 2.49–3.24 g kg^−1^ to 1.07–1.39 g kg^−1^ (relative reduction: 44.18–66.98%). Compared with the soil type-based grouping strategy, it further increased R^2^ from 0.70–0.74 to 0.81–0.85 (relative increase: 9.46–21.43%) and reduced RMSE from 1.91–2.56 g kg^−1^ to 1.07–1.39 g kg^−1^ (relative reduction: 27.23–58.20%). These outcomes suggest that conventional soil taxonomic information alone is insufficient to capture critical spectral variations. In the spectral grouping stage, DCAs were found to identify and aggregate soil samples with high spectral similarity more effectively than TCA. In particular, the inversion model constructed using Automatic Encoding Clustering (AEC) delivered the best performance. Additionally, the study revealed considerable spectral diversity, even among soils of the same type. In the evaluated dataset, spatial proximity was not consistently associated with spectral similarity. These findings confirm that spectral similarity-based grouping and modeling substantially improve the predictive accuracy of soil property estimation, offering a more refined and effective modeling pathway for hyperspectral soil detection. All spectral data analyzed here are laboratory-based Vis-NIR spectra from the national soil spectral library of China. These findings are based on the Chinese Soil Spectral Library and the Xinjiang dataset and should be validated in other regions and for other soil properties before broad application.

## 1. Introduction

Soil hyperspectral technology has been demonstrated to be an effective approach for the rapid and non-destructive assessment of soil properties [1,2,3,4,5]. This methodology commonly employs machine learning (ML) and deep learning (DL) algorithms to develop inversion models by analyzing the relationships between hyperspectral data and soil attributes [6]. Over the last two decades, substantial progress has been made in quantifying soil properties using hyperspectral techniques [5,7,8,9]. Conventional studies often focus on developing region-specific calibration models to predict soil properties for samples within the same geographic scope [10,11,12]. However, such models frequently exhibit limited transferability and poor generalization when applied to samples outside their original calibration regions [13,14]. In response, researchers have constructed soil spectral libraries (SSLs) encompassing diverse regions and soil types to calibrate large-scale global models for localized applications. Nevertheless, the outcomes remain suboptimal [9,15,16]. This limitation arises primarily from spatial heterogeneity in factors such as mineral composition, parent material, and climate across different regions, which results in divergent spectral characteristics. The resulting complex spectral variations challenge global modeling approaches in effectively capturing the intricate mapping relationships between spectral features and target soil properties [6]. Global models, by attempting to fit the spectral-property relationships across all samples, often incorporate interference from irrelevant spectral information present in heterogeneous samples, thereby compromising generalization capacity and predictive accuracy. To mitigate this issue, Alexandre et al. suggested that grouping similar soil samples by type prior to developing category-specific models can achieve better performance compared to global models-a conclusion supported by Ramirez-Lopez et al. [17].

Local modeling has become a well-established strategy for handling spectral heterogeneity in large soil spectral libraries. Memory-based learning (MBL) selects a subset of spectrally similar neighbors from a library for each target sample and constructs a local calibration model, and has been shown to outperform global models in multiple studies [17,18]. The spectrum-based learner (SBL) further improved local modeling by combining optimized principal component distances with Gaussian process regression. Nocita et al. developed a local partial least squares regression approach using geographically and spectrally selected neighbors. More recently, Viscarra Rossel et al. proposed a “think global, fit local” paradigm using transfer learning to adapt global models to local conditions. In parallel, spectral clustering has been explored as an alternative grouping strategy. Ramirez-Lopez et al. used K-means clustering to partition a Brazilian spectral library into subsets for improved prediction [19]. Previous studies have established local modeling and spectral grouping as effective strategies for handling soil spectral heterogeneity. Memory-based learning (MBL) selects a subset of spectrally similar neighbors for each target sample and builds a local calibration model [17,18]. The spectrum-based learner (SBL) further combines optimized principal component distance with Gaussian process regression [18]. Nocita et al. [10] developed a local partial least squares regression approach using geographically and spectrally selected neighbors. Viscarra Rossel et al. [14] proposed a “think global, fit local” transfer learning paradigm. In parallel, spectral clustering has been used to stratify soil spectral libraries. Ramirez-Lopez et al. [17] used K-means clustering to partition a Brazilian spectral library into subsets for improved prediction, and Ward et al. [20] applied spectral clustering to the LUCAS soil database for soil organic carbon prediction. These studies collectively show that local or grouped modeling can outperform global modeling when soil spectral heterogeneity is high. However, most previous studies have used a single clustering algorithm or a single family of algorithms, and a systematic comparison between traditional clustering algorithms (TCAs) and deep clustering algorithms (DCAs) for soil spectral partitioning is still lacking. In addition, the relationship between spectrally derived groups and conventional soil type classifications remains insufficiently quantified, and a reproducible workflow for assigning new samples to spectral clusters is rarely reported.

The main novelty of the present study is not the development of a new clustering algorithm or a new local modeling framework. Rather, it lies in the systematic comparison of five TCAs and five DCAs for spectral similarity-based grouping of the Chinese Soil Spectral Library (CSSL) supplemented with local Xinjiang samples, and in the explicit benchmarking of this grouping strategy against both traditional global modeling and soil type-based grouping for soil organic matter (SOM) prediction. We also provide an operational workflow for assigning new, unseen samples to spectral clusters. This workflow is not claimed as a methodological novelty; it is presented to make the group-level modeling strategy reproducible. Finally, we evaluate whether spatial proximity is consistently associated with spectral similarity and whether soil type labels alone are sufficient to capture spectral variation relevant to SOM prediction. These empirical and operational contributions distinguish the present study from previous spectral-grouping research. Finally, we describe an operational workflow for assigning new, unseen samples to spectral clusters and predicting their SOM content. This workflow is not claimed as a methodological novelty; it is provided to make the group-level modeling strategy reproducible and applicable. The main novelty of this study lies in the systematic comparison and benchmarking of spectral similarity-based grouping against global modeling and soil type-based grouping. Despite these advances, a systematic comparison between traditional clustering algorithms and deep clustering algorithms for soil spectral partitioning has not been conducted. Furthermore, the relationship between spectral groups derived from clustering and conventional soil type classifications remains insufficiently explored. Building on this idea, the present study proposes a spectral similarity-based grouped modeling strategy [21]. Unlike instance-based local modeling approaches such as memory-based learning (MBL) and the spectrum-based learner (SBL), which dynamically select spectral neighbors for each individual test sample and build a unique local model per prediction, the proposed strategy performs a one-time global partitioning of the entire spectral library into distinct groups based on spectral similarity [18]. A single specialized calibration model is then developed for each group, and all test samples assigned to that group are predicted using the corresponding group-level model. This group-level modeling framework offers a different perspective from instance-level local modeling: it produces a fixed, interpretable set of spectral groups, enables direct comparison of different clustering algorithms for spectral partitioning, and allows each group’s spectral characteristics to be independently analyzed and interpreted. This strategy aims to effectively minimize errors induced by soil sample heterogeneity and enhance prediction accuracy.

Selecting soil samples with similar spectral characteristics is essential for validating the hypotheses of this study. In recent years, alongside continuous advances in artificial intelligence (AI), traditional clustering algorithms (TCAs) have become a valuable tool in data analysis due to their strong ability to process unlabeled high-dimensional data [18,19,20,21,22]. By effectively capturing inter-sample feature distances, the TCA enables high-precision data grouping, ensuring high similarity among samples within the same cluster [23]. This capability provides a new perspective for efficiently identifying soil samples with comparable spectral profiles [13,20,21,22,23,24]. With the rapid development of deep learning (DL), many researchers have integrated DL with TCAs in fields such as medicine and signal recognition, leading to the emergence of deep clustering algorithms (DCAs) [25]. Studies have shown that, compared to traditional TCAs, DCAs can automatically extract higher-level features from raw data, making them more effective in handling high-dimensional, nonlinear, and complex-structured data, thereby improving clustering accuracy and robustness [25]. However, the application of TCAs in soil hyperspectral analysis remains in its early stages. Although some studies have explored the use of traditional clustering methods, such as k-means and hierarchical clustering on soil spectral data, a systematic comparison between traditional TCAs and DCAs in terms of their effectiveness in soil spectral applications has yet to be conducted [6,13,20,23]. This gap not only limits our understanding of how clustering algorithms can be applied to soil hyperspectral data but also complicates the selection of the most suitable clustering strategy for soil spectral studies. These challenges motivate further in-depth investigation into this topic.

In summary, this study will validate the proposed hypothesis using the Chinese Soil Spectral Library (CSSL) along with localized data from the Xinjiang Uygur Autonomous Region. The dataset consists of 3700 entries spanning 22 provinces and encompassing 16 soil types. To thoroughly assess the feasibility of enhancing soil organic matter (SOM) prediction accuracy, ten clustering algorithms, including five traditional clustering algorithms (TCAs) and five deep clustering algorithms (DCAs), will be employed to group soil samples based on spectral similarity, with separate inversion models developed for each group. Furthermore, to provide a comparative basis, soil samples will also be categorized by soil type using CSSL data, and corresponding models will be established as a benchmark. The general objective of this study is to systematically evaluate and compare the effectiveness of spectral similarity-based clustering strategies versus traditional soil type-based grouping strategies in mitigating the technical failure caused by soil unevenness in Vis-NIR spectroscopic prediction of SOM. Based on the above, we propose the following testable hypotheses: (1) Spectral similarity-based grouping improves SOM prediction accuracy compared with traditional global modeling; (2) Spectral similarity-based grouping improves SOM prediction accuracy compared with soil type-based grouping; (3) Deep clustering algorithms (DCAs) produce more spectrally homogeneous groups and yield better SOM prediction accuracy than traditional clustering algorithms (TCAs); (4) Spatial proximity among soil samples is not necessarily associated with spectral similarity, and soil type labels alone are insufficient to capture spectral variation relevant to SOM prediction.

## 2. Materials and Methods

### 2.1. China Soil Spectral Library

**Figure 1:**
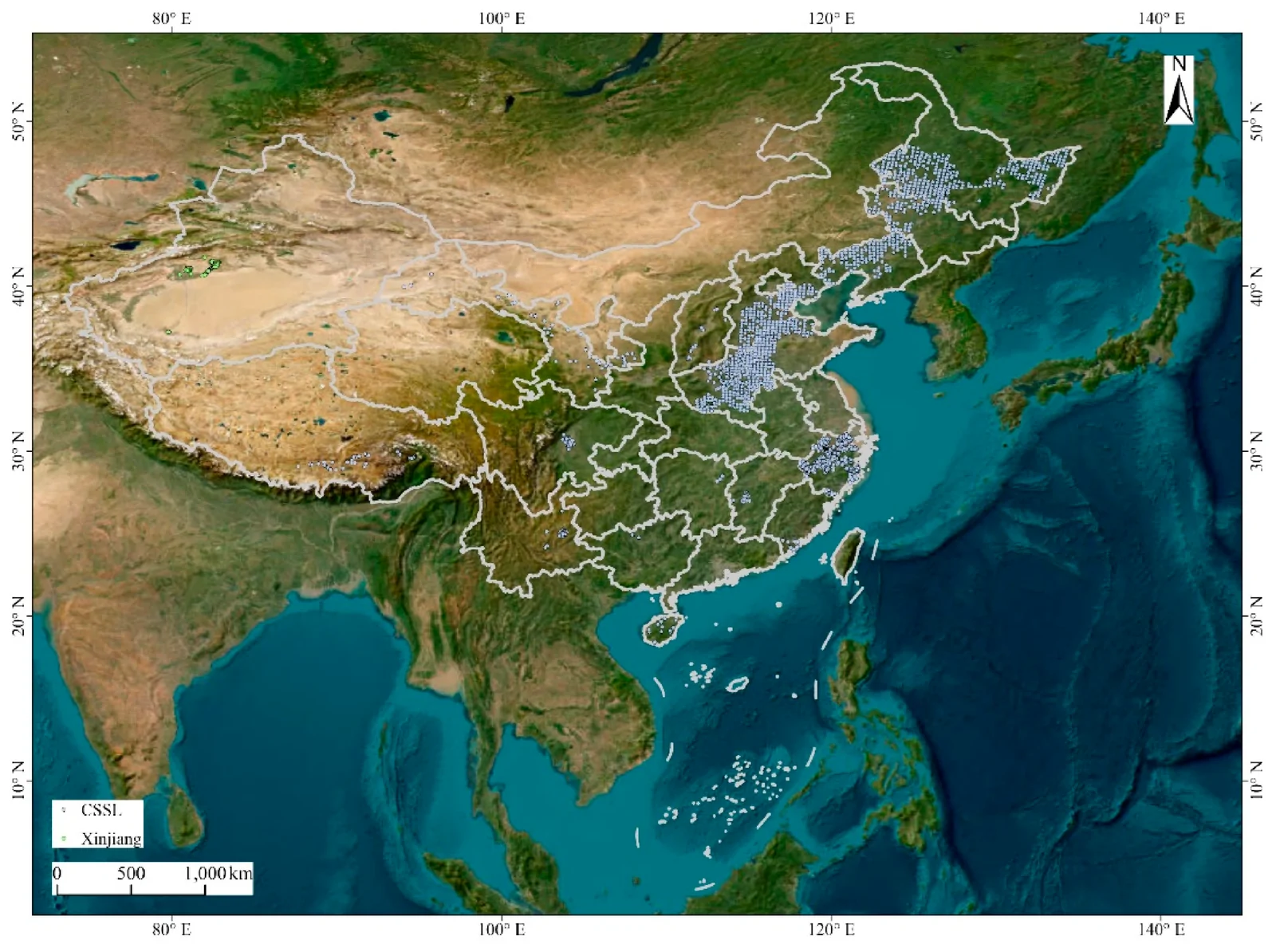
Sampling distribution map of the soil spectral library in China. In the picture, the green dots representing the research area indicate the locations of the sample points in Xinjiang, while the blue dots represent the distribution of sample points from various provinces in China, sourced from the Chinese Soil Spectral Database.

The development of the Chinese Soil Spectral Library (CSSL) was initiated under the leadership of Professor Shi Zhou from Zhejiang University, with collaborative efforts from multiple research teams. In 2010, a total of 1581 soil samples were collected across 14 provinces in temperate and cold regions of China. These samples covered 16 soil types, including meadow soils, black soils, Chernozems, and paddy soils from southern China. All soil properties were determined through standardized chemical laboratory analyses [13]. To date, the CSSL has expanded to include 3000 samples, establishing itself as China’s largest open-source soil spectral database [24]. However, the CSSL originally lacked representation from the Xinjiang Uygur Autonomous Region. Located in a typical arid inland zone, Xinjiang exhibits a distinctive natural environment characterized by extreme aridity, high evaporation rates, and widespread soil salinization, which collectively give rise to region-specific soil types [26]. To enhance the comprehensiveness and national representativeness of the Chinese soil spectral database, this study collected an additional 700 soil samples from the Tarim River Basin in Xinjiang, thereby incorporating this critical regional dataset into the CSSL. Figure 1 and Table 1 present the spatial distribution of the CSSL and Xinjiang samples, along with descriptive statistics of soil organic matter (SOM) content by soil type. Note that due to the limited sample sizes of Alluvial soils, Latosols, and Aeolian soils, these types will be excluded from subsequent soil-type-specific modeling to ensure analytical validity. The sampling areas primarily encompass oasis farmland regions dominated by economic crops such as cotton and jujube, where drip irrigation under plastic mulch is the prevailing agricultural practice [8]. Sample selection took into account key environmental factors such as topography, parent material, soil type, and climatic characteristics [13]. The inclusion of these samples not only fills a critical gap in the CSSL concerning arid zone soils but also provides an essential data foundation for investigating the spectral response mechanisms of such soils and enabling high-accuracy inversion of soil properties. This effort holds significant value for improving the generalizability of national-scale soil spectral models and supporting precision agricultural management in arid regions. Laboratory-based visible–near-infrared (Vis-NIR) spectra were acquired for all soil samples. For the CSSL samples, spectra were measured using an ASD FieldSpec 4 Hi-Res spectroradiometer (Malvern Panalytical Ltd., Malvern, UK) over the 350–2500 nm range. The spectral sampling interval was 1.4 nm in the 350–1000 nm region and 2 nm in the 1000–2500 nm region, with a spectral resolution of 3 nm at 700 nm and 10 nm at 1400 nm and 2100 nm. Samples were air-dried, gently ground, and passed through a 2 mm sieve. Each sample was placed in a Petri dish and measured under a halogen lamp with a contact probe. A white reference panel was used for calibration before each measurement, and the spectra were recorded as reflectance. For the Xinjiang local samples, the same instrument and measurement protocol were used to ensure consistency with the CSSL data. Detailed measurement procedures for the CSSL and the Xinjiang dataset are also provided in [5,13,27].

It should be noted that the sample sizes of the remaining soil types were also uneven. For example, black soils and paddy soils had relatively large sample sizes, while some other soil types had fewer samples. This imbalance is common in national soil spectral libraries and can affect the performance of soil type-based grouping models. We have acknowledged this limitation in the discussion. Soil organic matter (SOM) content was determined by the dichromate oxidation method (or the method actually used). For the CSSL samples, SOM was measured using the standardized procedures described in Shi et al. [13]. For the Xinjiang samples, the same analytical protocol was followed to ensure comparability. All spectral measurements were performed with the same instrument model (ASD FieldSpec 4 Hi-Res) and the same measurement protocol. Therefore, the CSSL and Xinjiang spectra were harmonized, and no inter-instrument standardization was required.

### 2.2. Spectral Preprocessing

Savitzky–Golay (SG) smoothing and denoising and the Standard Normalization Variation (SNV) are two techniques commonly used for preprocessing visible–near-infrared (Vis-NIR) spectral data from samples [28,29]. SG reduces noise in spectral data by fitting data points in local intervals using polynomial curves. SNV eliminates variations between samples caused by differing measurement conditions by mean-centering and normalizing each spectrum [28]. Research indicates that SG and SNV processing effectively enhances spectral data quality, establishing a solid foundation for spectral analysis and modeling [30]. Therefore, this study employs SNV and SG for spectral preprocessing. The SG smoothing window size was set to 11, and the polynomial order was set to 2 (i.e., a second-order polynomial, not zero). The choice of SG parameters was based on both the spectral resolution of the data and common practice in soil Vis-NIR preprocessing. The preprocessing sequence was as follows: first, Savitzky–Golay smoothing (window size = 11, polynomial order = 2) was applied to reduce high-frequency noise; second, standard normal variate (SNV) transformation was applied to remove multiplicative scatter effects; third, the spectra were standardized using z-score normalization (zero mean and unit variance) before clustering and modeling. A window size of 11 corresponds to approximately 11–22 nm across the 350–2500 nm range, depending on the sampling interval. This window is wide enough to suppress high-frequency noise while narrow enough to preserve the shape and position of absorption features. A second-order polynomial was selected because it can capture the curvature of spectral curves without overfitting local noise. A zero-order polynomial (moving average) would over-smooth the spectra and weaken absorption features, while higher orders could introduce oscillations. These parameters are consistent with previous soil spectral studies [28,29,30]. Furthermore, prior research indicates that utilizing full-spectrum data during exploratory analysis of spectral similarity provides richer spectral information, thereby enhancing classification accuracy [31,32,33]. Consequently, this study will employ full-spectrum data for subsequent analyses.

### 2.3. Traditional Clustering Algorithm

**Figure 2:**
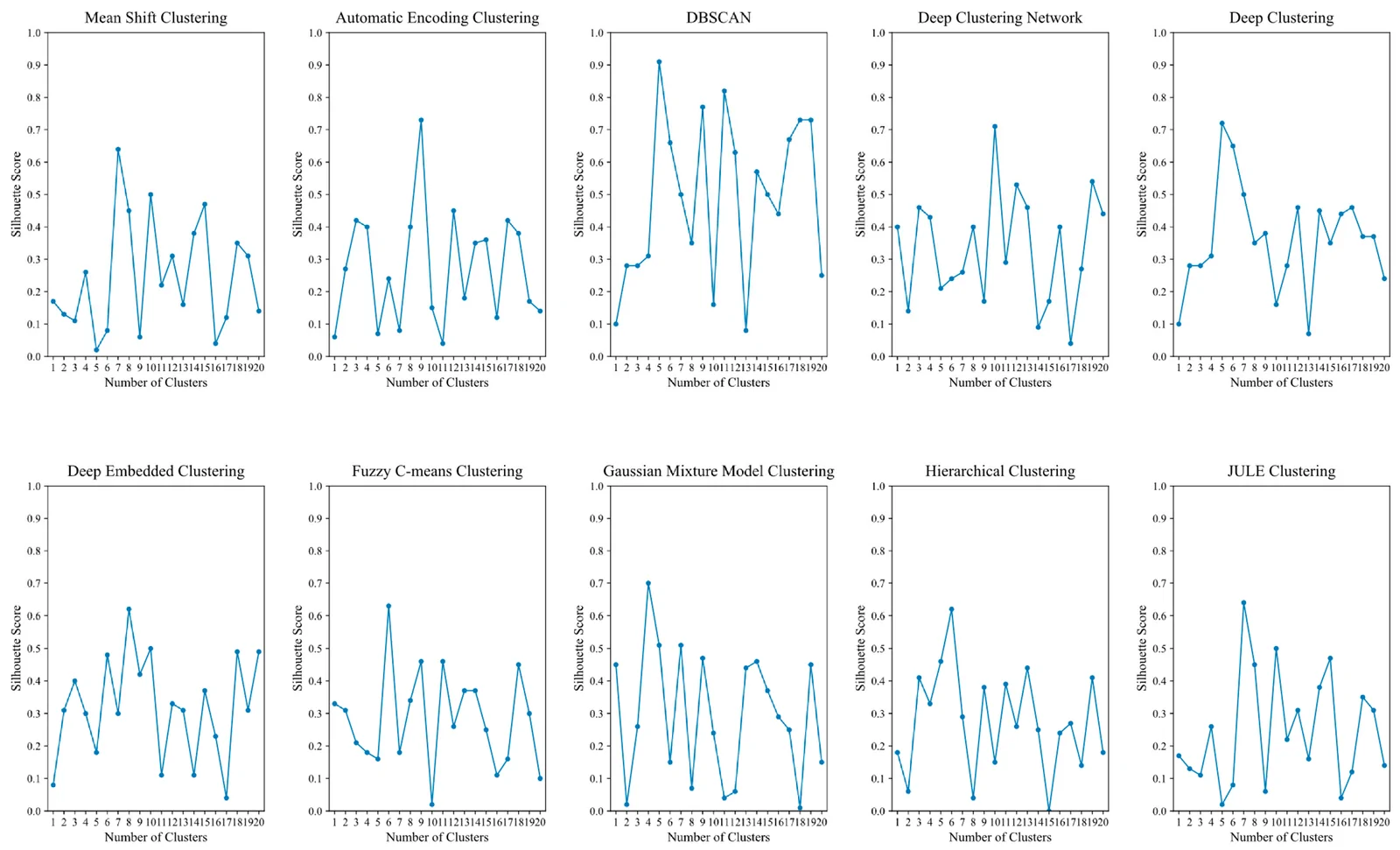
The optimal number of clusters for different clustering algorithms is selected based on the contour coefficient. The contour coefficients in the figure represent the size of the coefficient of variation for each clustering method when the number of clusters ranges from 1 to 20.

**Figure 3:**
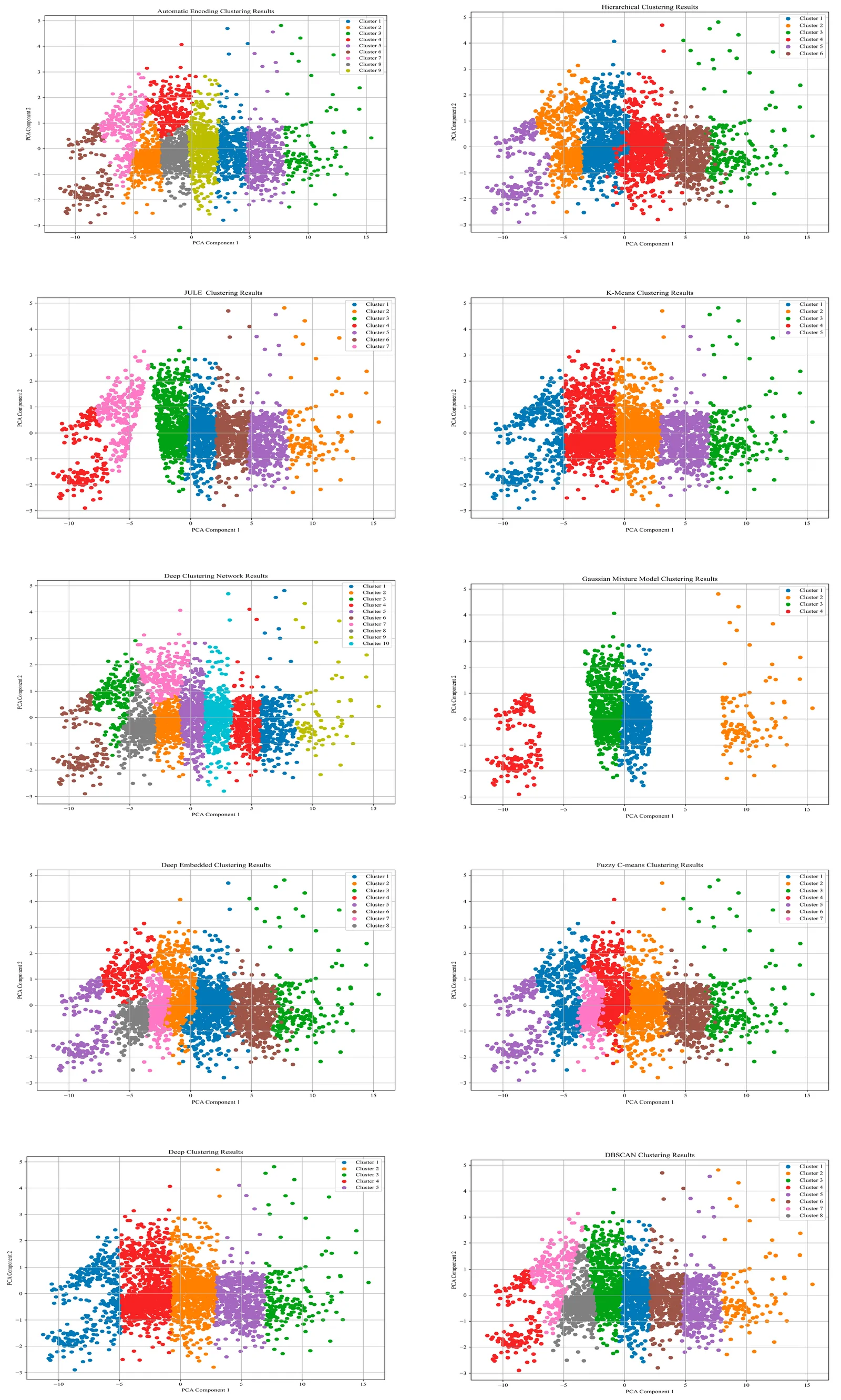
Cluster results of different clustering algorithms. The figure shows the spectral characteristics of each cluster after clustering by the brother clustering algorithm, presented in a two-dimensional plane after dimensionality reduction.

**Figure 4:**
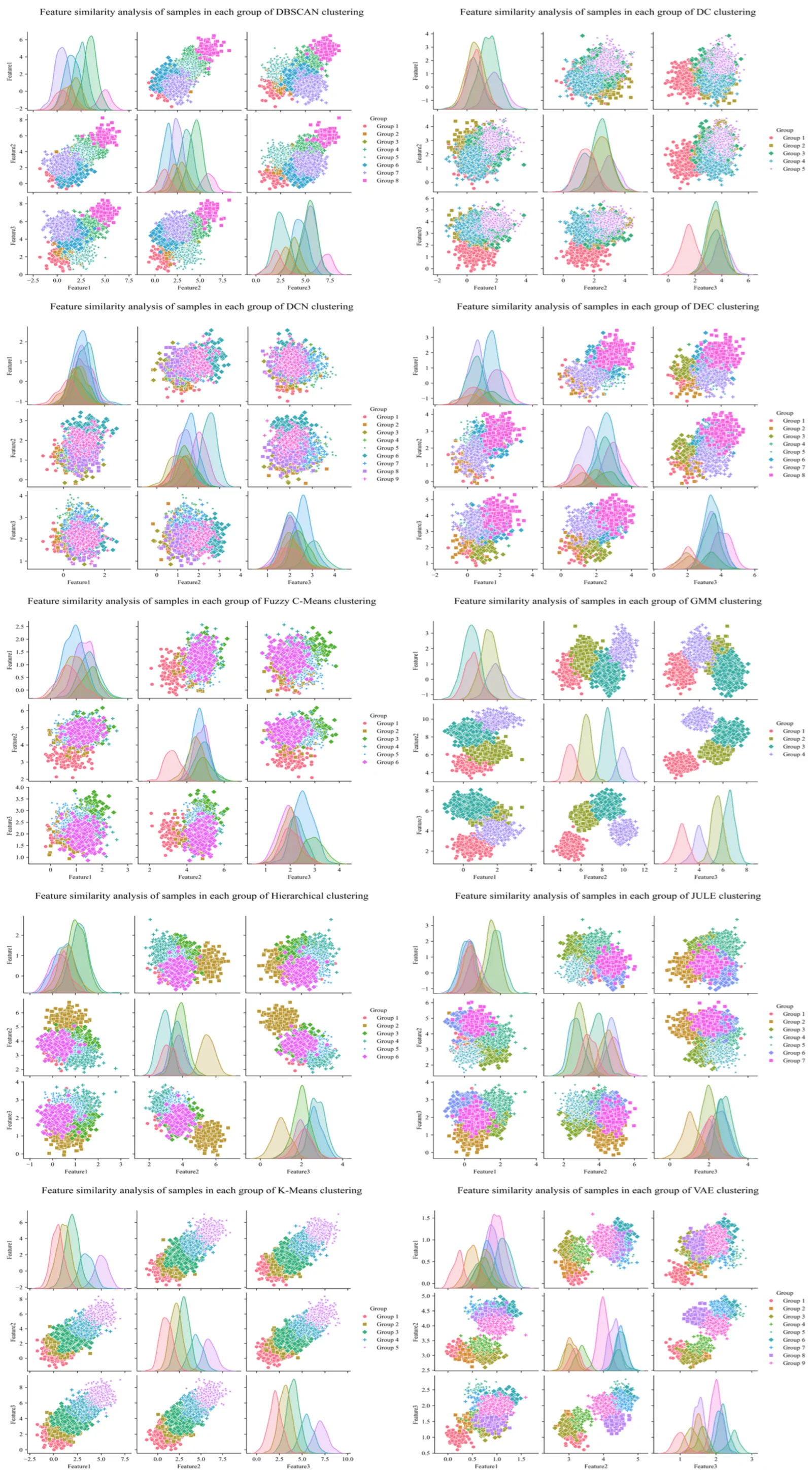
Similarity of spectral features of different soil clusters.

**Figure 5:**
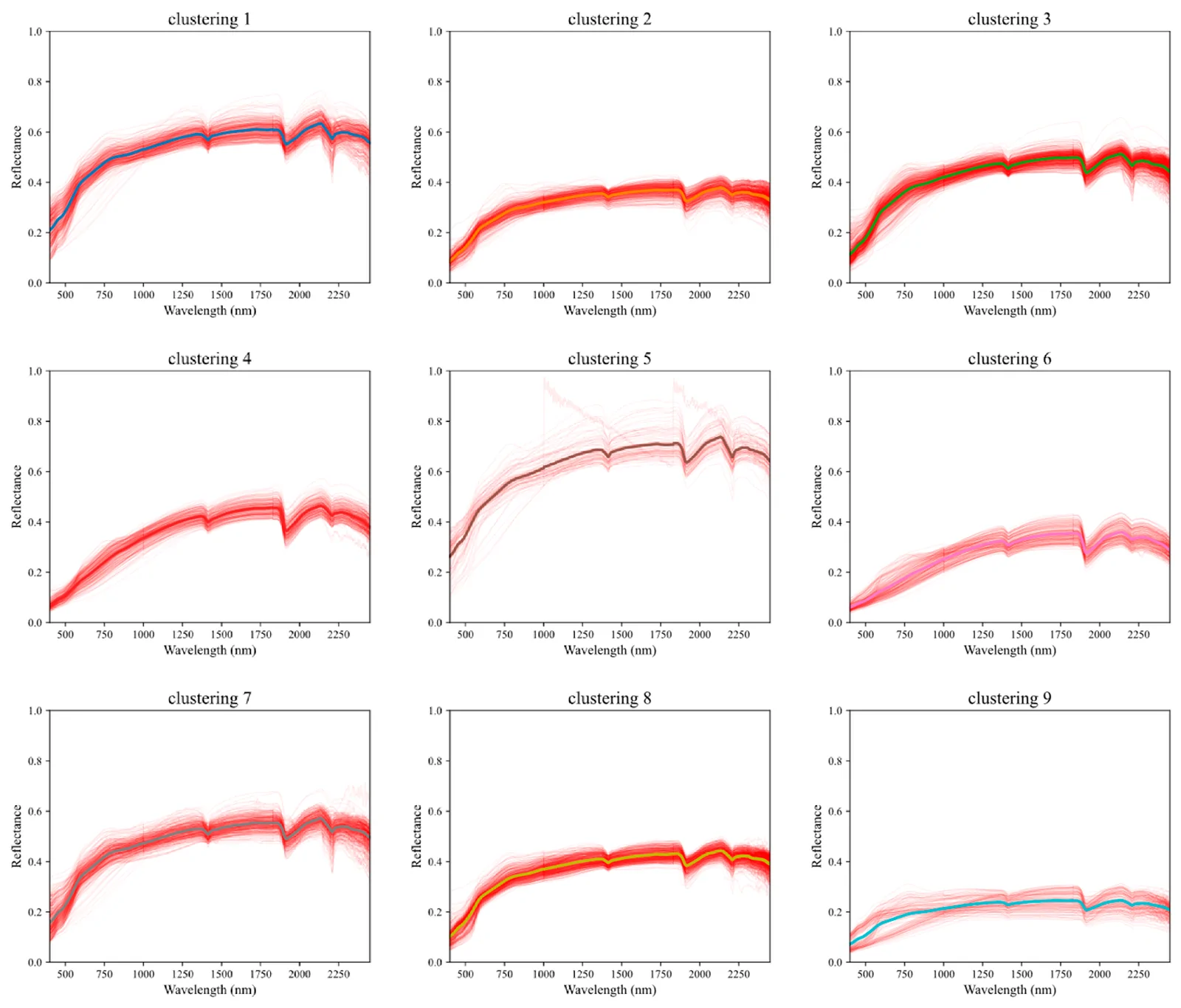
The spectral curves of each cluster after AEC.

**Figure 6:**
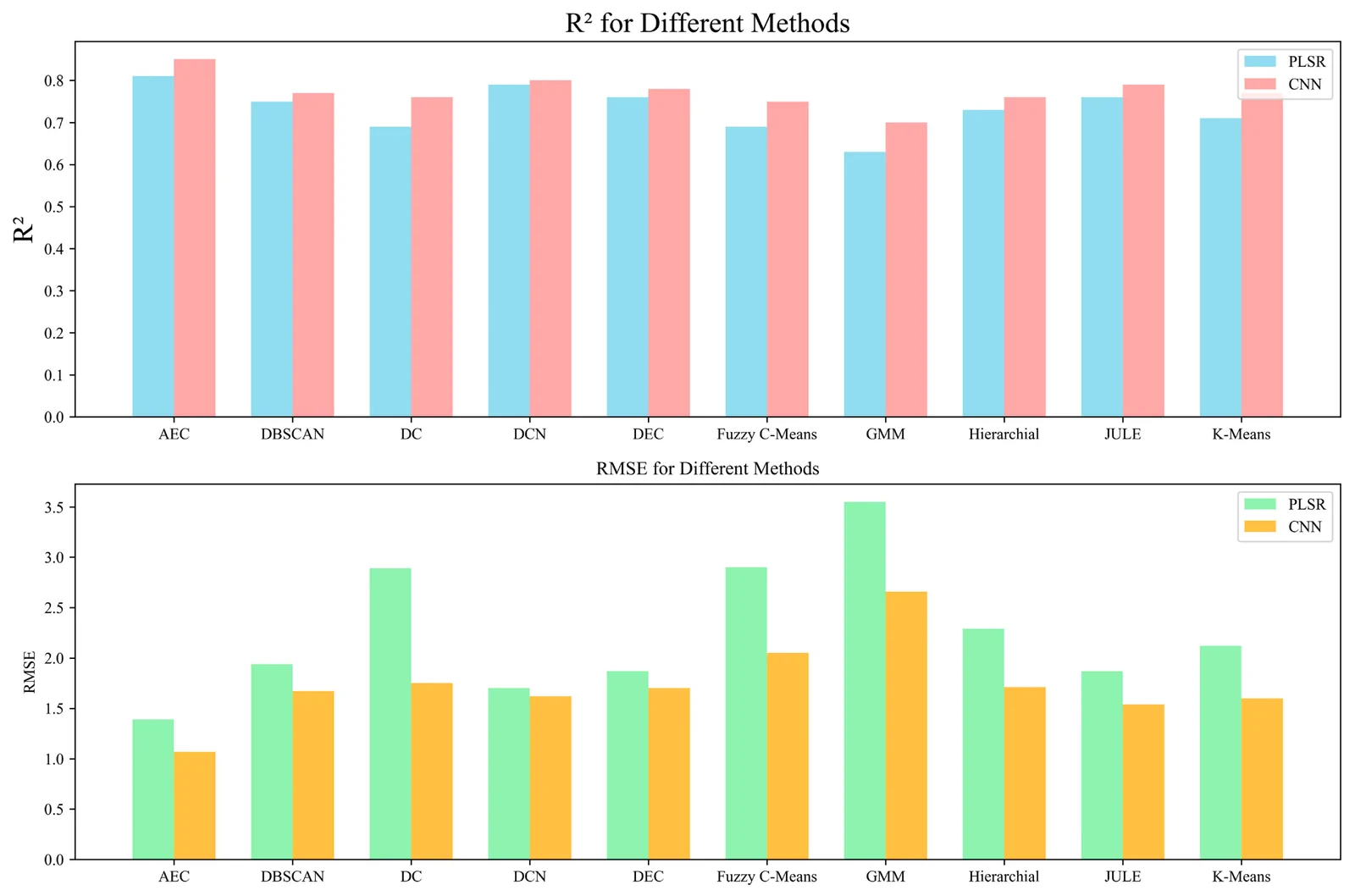
After grouping by different clustering methods, the average performance of each clustering group sample modeled in the test set was calculated. Here, R^2^ represents the fitting degree, and RMSE (g kg^−1^) represents the error. Each clustering method separately shows the average performance of the PLSR and CNN models established for each group in the validation set.

Clustering is an unsupervised learning technique widely applied in data analysis, market segmentation, image processing, and social network analysis [34,35]. Clustering algorithms aim to group objects within a dataset into distinct clusters based on feature similarity [26,35]. This study will select five clustering algorithms commonly used in the field of spectroscopy to analyze the spectral similarity of soil samples. These include K-Means, Fuzzy C-Means (F-C-Means), Density-Based Clustering (DBSCAN), Gaussian Mixture Model Clustering (GMMC), and Hierarchical Clustering (HC) [13,23,36,37,38,39]. Schematic diagrams of the five clustering methods are provided (see Figure 2, Figure 3, Figure 4, Figure 5 and Figure 6).

For the clustering analysis of soil visible–near-infrared (Vis-NIR) spectral data, this paper employed five clustering algorithms based on different principles. All algorithms were implemented using the Scikit-learn library in the Python 3.13.0 environment. The fuzzy C-means clustering was implemented using the scikit-fuzzy library. Before applying the clustering algorithms, the original spectral data were standardized using z-score normalization (zero mean and unit variance) to eliminate the influence of differences in the scale of each band on the distance calculation. Standardization was applied for three reasons. First, soil Vis-NIR spectra contain hundreds of continuous bands, and the reflectance magnitude and variance differ substantially among bands. Without standardization, bands with larger numerical ranges would dominate the distance calculation in clustering algorithms, while bands with smaller ranges would contribute little. This would distort the clustering results and prevent the algorithms from grouping samples based on overall spectral shape. Second, the CSSL samples and the Xinjiang samples were collected over different periods. Although the same instrument model (ASD FieldSpec 4 Hi-Res) and the same measurement protocol were used for all samples, minor systematic differences due to measurement date or environmental conditions may still exist. Z-score normalization reduces these potential differences and ensures that clustering is based on spectral shape rather than absolute reflectance. Third, standardization is a standard and necessary preprocessing step for distance-based clustering algorithms. The same z-score normalization was applied to all ten clustering algorithms to ensure a fair comparison. The standardization parameters (mean and standard deviation) were computed from the training set only and then applied to the test set to avoid data leakage. Standardization was applied for three reasons. First, soil Vis-NIR spectra contain hundreds of continuous bands, and the reflectance magnitude and variance differ substantially among bands. Without standardization, bands with larger numerical ranges would dominate the distance calculation in clustering algorithms, while bands with smaller ranges would contribute little. This would distort the clustering results and prevent the algorithms from grouping samples based on overall spectral shape. Second, the CSSL samples and the Xinjiang samples were collected over different periods. Although the same instrument model (ASD FieldSpec 4 Hi-Res) and the same measurement protocol were used for all samples, minor systematic differences due to measurement date or environmental conditions may still exist. Z-score normalization reduces these potential differences and ensures that clustering is based on spectral shape rather than absolute reflectance. Third, standardization is a standard and necessary preprocessing step for distance-based clustering algorithms. The same z-score normalization was applied to all ten clustering algorithms to ensure a fair comparison. The standardization parameters (mean and standard deviation) were computed from the training set only and then applied to the test set to avoid data leakage. Figures S1–S5 respectively show the structural diagrams of K-Means, fuzzy C-means, DBSCAN, Gaussian Mixture Model, and hierarchical clustering, clearly presenting the iterative process, membership update rules, density-connected determination, probability distribution fitting, and tree-like aggregation logic of each algorithm. For details, please see Figures S1–S5 in the Supplementary Materials.

For K-Means, the Euclidean distance was used as the distance metric, which is appropriate because all spectral bands were standardized to zero mean and unit variance before clustering. The number of clusters k was searched from 1 to 20 using the silhouette coefficient, and the k that maximized the average silhouette coefficient was selected. The random state was fixed at 42 for reproducibility. For Fuzzy C-Means, the fuzzy index m was set to 2, which is a commonly used value in spectral clustering. The maximum number of iterations was 1000, and the tolerance was 0.005. The number of clusters c was searched from 1 to 20 using the silhouette coefficient, following the same procedure as K-Means. For DBSCAN, the epsilon parameter was determined using the k-distance graph, and min_samples was set to 5 to ensure that each core cluster contained at least five soil samples. The silhouette coefficient was also used to evaluate the clustering quality across a range of epsilon values. This procedure ensured that the density threshold was not arbitrary but adapted to the standardized spectral data. For Gaussian Mixture Model clustering, the number of components was searched from 1 to 20 using the silhouette coefficient. Four covariance types (full, tied, diag, and spherical) were compared, and the full covariance type was selected because it provided the best clustering quality. The random state was fixed at 42. For Hierarchical Clustering, the Ward variance minimization method was used as the linkage criterion, and Euclidean distance was used as the distance metric. The dendrogram was cut using a distance threshold of 1.5, and the number of clusters was jointly evaluated using the silhouette coefficient within the range of 1 to 20. It should be explicitly noted that the five traditional clustering algorithms were applied directly to the standardized full-spectrum data, without additional dimensionality reduction such as PCA, UMAP, or wavelet transform. This choice was made because the objective of this study is to evaluate the inherent capability of each clustering algorithm to group soil samples based on spectral similarity in the original spectral space. The deep clustering algorithms described in Section 2.4 also received the same standardized full-spectrum data as input; their encoders learned low-dimensional representations as part of the model, rather than as a separate preprocessing step. Therefore, all algorithms started from the same input data, and the comparison reflects the different ways in which traditional and deep clustering algorithms handle high-dimensional spectral information.

### 2.4. Deep Clustering Algorithm

The Deep Clustering Algorithm (DCA) integrates deep learning techniques with traditional clustering algorithms (TCAs), leveraging deep neural networks to achieve more accurate and efficient clustering [40,41]. A key feature of DCAs is their ability to learn complex feature representations of data, perform nonlinear mapping and feature extraction on high-dimensional data, thereby better capturing the inherent structural information within the data and improving clustering accuracy and robustness [42,43,44]. This study selects five commonly used DCAs to explore their performance in soil spectral analysis: Deep Embedded Clustering (DEC), Autoencoder Clustering (AEC), Deep Clustering (DC), Deep Clustering Network (DCN), and Joint Unsupervised Learning of Deep Representations and Image Clusters (JULE). Structural diagrams of the five DCAs. DEC extracts features and performs clustering through deep learning (DL) networks, enabling capture of complex data structures but exhibiting sensitivity to initialization parameters and hyperparameter settings [45]. AEC combines the strengths of autoencoders (AE) and clustering, handling high-dimensional data to generate robust clustering results [46,47]. DC effectively integrates deep feature learning with K-means clustering through iterative optimization, enhancing clustering performance [48]. DCN simultaneously learns deep feature representations and clustering structures through iterative optimization strategies [49]. JULE concurrently optimizes feature representations and clustering outcomes, adapting to diverse data scales. However, its complex architecture demands additional computational resources and iterative hyperparameter tuning [50].

All algorithms were implemented in Python 3.13.0 based on the TensorFlow/Keras framework, and the network structure was adapted to the one-dimensional sequence characteristics of soil spectra. In the data preprocessing stage, the original spectral reflectance (covering the visible to near-infrared bands) was standardized (using StandardScaler) to eliminate the influence of dimensionality. To fairly evaluate the clustering quality, all deep clustering algorithms searched for the optimal number of clusters within the range of 1 to 20 by using the silhouette coefficient; that is, running the corresponding model for each possible cluster number and calculating the silhouette coefficient, selecting the cluster number corresponding to the maximum value as the final clustering number to avoid bias caused by artificially presetting the number of clusters.

The structure diagram of DEC clearly shows the collaborative relationship between the encoder, embedding space, and clustering module. DEC first defines an autoencoder structure consisting of an encoder, decoder, and K-Means. The encoder is composed of multiple fully connected (Dense) layers and is responsible for compressing the high-dimensional spectral input into a low-dimensional embedding representation. The key parameter encoding_dim is set to 128; each encoding layer uses the ReLU activation function to alleviate gradient vanishing, and the decoder uses the sigmoid activation function to ensure that the output values are limited to the [0, 1] interval to match the standardized spectral reflectance. During model compilation, the Adam optimizer and mean squared error (MSE) loss function are used, and the model is trained using the fit method with epochs = 500 and batch_size = 256. After training, the decoder is discarded, and only the trained encoder is used to extract the 128-dimensional embedding features of all soil samples, and then K-Means clustering is performed in this embedding space (while selecting the optimal number of clusters within 1–20 by using the silhouette coefficient). For all deep clustering algorithms, the key hyperparameters (including the embedding dimension, learning rate, batch size, and number of training epochs) were selected through a grid search on a validation subset of the training data. The silhouette coefficient was used to determine the optimal number of clusters for each algorithm within the range of 1 to 20. The same standardized spectral data and the same evaluation metric were used for both traditional and deep clustering algorithms to ensure a fair comparison.

The structure of AEC is similar to DEC, and its structure diagram shows a more prominent reconstruction branch of the decoder, indicating that this algorithm focuses on high-fidelity reconstruction without introducing clustering loss; the parameter configuration is exactly the same as DEC (encoding_dim = 128, epochs = 500, batch_size = 256, Adam, MSE), and the embedding features are extracted, and K-Means clustering is performed after the autoencoder training is completed. Although AEC shares a similar autoencoder backbone with DEC, its training strategy is fundamentally different. DEC jointly optimizes reconstruction and clustering losses in an end-to-end manner. In contrast, AEC is trained in two separate stages: first, the autoencoder is trained purely for reconstruction; second, K-Means clustering is performed on the learned embedding features. No clustering loss is used during autoencoder training. This decoupled design avoids the conflicting optimization between reconstruction and clustering, and it is one reason why AEC performed better than DEC in our experiments.

For convolution-based deep clustering algorithms, the structure diagram of DCN emphasizes the “feature extraction-clustering assignment-joint optimization” three parts: first, the standardized spectral data is normalized, and then one-dimensional convolutional neural network (CNN) is used to extract features, which contains a convolution layer (128 channels, with a convolution kernel size of 3 to capture local spectral absorption patterns) and a max pooling layer; the extracted features are then mapped to a new space by initializing the cluster centers and assigning each sample to the nearest center, calculating the clustering loss, and finally, the network parameters and cluster center positions are jointly optimized through backpropagation.

The structure diagram of DC is similar to DCN, using a one-dimensional CNN to extract features (128 channels), but adding a fully connected layer. The features are mapped to a new space through a nonlinear activation function, and the reconstruction loss and clustering loss are calculated simultaneously; finally, the network parameters and cluster centers are jointly optimized through backpropagation.

The structure diagram of JULE presents a recursive aggregation tree-like structure. Its core idea is to iteratively merge the nearest neighbor samples in the deep feature space to construct a clustering binary tree (tree diagram), and each merge is adjusted by backpropagation to make the feature representation continuously adapt to the current clustering partition; in this algorithm, the embedding dimension is also set to 128, the default tree depth is 5 layers, and the merging threshold is determined through the validation set.

The above algorithms were implemented in an environment with the operating system set to Ubuntu 22.04 LTS and the Python version set to 3.13.0. The key dependent libraries and their exact version numbers include: numpy 1.26.2, scipy 1.11.4, pandas 2.1.3, scikit-learn 1.3.2, scikit-fuzzy 0.4.2, tensorflow 2.15.0 (with GPU support enabled), keras 2.15.0. Additionally, CUDA 12.1 and cuDNN 8.9.4 were used, and the commands for installing the same environment using pip and conda are provided. Secondly, we systematically controlled all sources of randomness: not only did we set the Python built-in random number seed as random.seed (42), the NumPy global seed as np.random.seed (42), and the TensorFlow seed as tf.random.set_seed (42).

To ensure a fair comparison between traditional and deep clustering algorithms, all methods were applied to the same z-score standardized spectral data. For each algorithm, the key parameters were either determined by established rules (e.g., k-distance graph for DBSCAN) or selected by searching a reasonable range using the silhouette coefficient (e.g., number of clusters for K-Means, FCM, GMM, and HC). For deep clustering algorithms, the network architecture and training parameters were selected through a grid search on a validation subset. No algorithm was run with arbitrary or default parameters without justification. The same evaluation procedure was applied to all methods, so that the comparison reflects the inherent capability of each algorithm to produce spectrally homogeneous groups. For the deep clustering algorithms, the standardized full-spectrum data were used as input, and the dimensionality reduction was performed internally by the encoder. No external dimensionality reduction method was applied before the deep clustering algorithms either, so that the comparison with traditional clustering algorithms is based on the same original spectral input. Figures S6–S10. For details, please refer to the attached figure.

For all deep clustering algorithms, the encoders were trained using only the 70% training data. A validation subset was split from the training data, and all key hyperparameters, including the embedding dimension, learning rate, batch size, and number of training epochs, were selected through a grid search on this training-only validation subset. The number of clusters was searched from 1 to 20 using the silhouette coefficient computed on the training data only. The independent test set was never used for hyperparameter selection, early stopping, or cluster-number selection.

### 2.5. The Method of Determining the Optimal Number of Clusters

For all ten clustering algorithms, the number of clusters was selected using the silhouette coefficient computed exclusively on the 70% training set. The independent test set was not used at any stage of cluster-number selection. For methods requiring additional parameters, those parameters were either fixed at standard values for reproducibility or selected using the training data only. No parameter was tuned using the independent test set. The silhouette coefficient is a metric that can be used to determine the optimal number of clusters for all clustering algorithms [51]. Unlike the elbow rule and dendrogram, which are only applicable to K-Means and hierarchical clustering (HC), the silhouette coefficient can be used to determine the optimal number of clusters for various clustering algorithms [52,53]. The silhouette coefficient assesses cluster quality by measuring how tightly data points cluster together within their own cluster while maintaining separation from other clusters [51]. The silhouette coefficient ranges from −1 to 1, where SC > 0 and close to 1 indicates data points are well clustered together and distinctly separated from other clusters; SC < 0 indicates data points are misclassified into inappropriate categories. The silhouette coefficient is calculated as follows: For each data point (i), first compute its average distance within its own cluster (a(i)), defined as the mean distance from point (i) to all other points in the same cluster. Next, calculate its average distance to the nearest neighboring cluster (b(i)), defined as the mean distance from point (i) to the closest point in another cluster. The silhouette coefficient (s(i)) can then be computed using the following formula:

(1) $$s(i)=\frac{b(i)-a(i)}{\max\{a(i),b(i)\}}$$

### 2.6. Expected Modeling Algorithm

To further investigate the accuracy of clustering algorithms and the predictive performance of models based on clustered samples, two widely recognized models in the spectral domain were employed to establish separate models for each sample group: Partial Least Squares Regression (PLSR) and Convolutional Neural Networks (CNNs) [54,55]. These models were selected because PLSR and CNN have been demonstrated in multiple studies to be optimal for capturing both linear and nonlinear relationships between soil properties and soil spectral characteristics [9,56,57,58]. By analyzing the predictive performance of PLSR and CNN, the feasibility of this approach can be explored more comprehensively and accurately. The 1D-CNN was constructed using the TensorFlow library, comprising four convolutional layers with filter counts of 16, 32, 64, and 128, respectively. The convolution kernel size was set to 3, the activation function to “Linear,” and the pooling layer window size to 2. Training employed the Adam optimizer with mean squared error (MSE) as the loss function.

To address the potential risk of overfitting when training CNN models on individual clusters, we evaluated the sample size of each cluster before modeling. The smallest cluster contained more than 80 samples, and the median cluster size was around 300 samples. To further prevent overfitting, we applied several regularization strategies during CNN training: (1) dropout with a rate of 0.2 after each convolutional block; (2) L2 weight decay with a coefficient of 1 × 10^−4^; (3) early stopping with a patience of 20 epochs based on the validation loss; and (4) data augmentation by adding Gaussian noise (signal-to-noise ratio of 30 dB) and random wavelength shifts (±2 nm) to the training spectra. In addition, the CNN architecture was intentionally kept compact, with only four convolutional layers and a small number of filters, to reduce the number of trainable parameters. These measures effectively controlled overfitting and ensured that the CNN models generalized well to the test samples.

### 2.7. Data Division and Evaluation Index

This study employs three modeling schemes: traditional global modeling, soil type-based grouping modeling, and spectral similarity-based grouping modeling. To ensure a strictly fair comparison among all schemes and all clustering algorithms, the complete dataset was first partitioned into 70% training and 30% independent testing subsets using the Kennard If more than one instrument Stone (K–S) algorithm. This split was performed once and fixed for all subsequent analyses. The 30% independent test set was held out before any standardization, clustering, representation learning, or model development, and was never used to define cluster boundaries or to select hyperparameters. Standardization parameters (mean and standard deviation) were computed only from the training set and then applied to the test set.

For the spectral similarity-based scheme, each of the ten clustering algorithms was trained using only the 70% training data. For deep clustering algorithms, the encoders were trained only on the training data, and all hyperparameters were selected using a validation subset of the training data. After clustering, a separate PLSR and CNN model was trained for each cluster using only the training samples assigned to that cluster. The 30% independent test samples were then assigned to the existing clusters using clearly defined out-of-sample rules. Each test sample was predicted using the model of the cluster to which it was assigned. Finally, all predictions from the independent test set were pooled to compute global R^2^ and RMSE values. Because every clustering method was evaluated on exactly the same independent test samples, the comparison among methods is strictly fair.

Assessing similarity among clustered sample groups is a crucial method for evaluating clustering algorithm performance. By examining feature similarity within groups, one can intuitively determine whether spectral characteristics of samples within a group are comparable [33,59]. Principal Component Analysis (PCA) is the most commonly used method for sample similarity testing [60,61,62]. PCA first standardizes the data within each sample group to have a mean of 0 and variance of 1. It then calculates the covariance matrix, performs an eigenvalue decomposition, and selects several components that explain the majority of the variance. Finally, the data is projected onto these principal components, and the projected data can be visualized to observe the degree of overlap along the principal components, thereby assessing spectral feature similarity. Additionally, another method to evaluate clustering accuracy involves establishing inversion models for each sample group and quantifying the clustering algorithm’s precision by assessing the inversion models’ performance [33]. The principle behind establishing inversion models is to capture the relationship between soil properties and spectral characteristics. If spectral similarity is high within a sample group, the model can more accurately capture this relationship. This study uses the coefficient of determination (R^2^) and root mean square error (RMSE) to evaluate inversion model performance. Generally, an excellent model should exhibit a high R^2^ value and a low RMSE value. RMSE measures the standard deviation of errors between observed values and model predictions. A smaller RMSE indicates greater predictive accuracy. R^2^ assesses the model’s fit to observed values. With values ranging from 0 to 1, an R^2^ closer to 1 signifies a better fit to the data [63].

Model assignment for new samples. In the proposed workflow, a new, unseen sample must first be assigned to one of the spectral groups before its SOM content can be predicted using the corresponding group-level model. We therefore defined explicit out-of-sample assignment rules for each clustering algorithm. For K-Means, the new sample was assigned to the cluster whose centroid was closest in the standardized spectral space. For Fuzzy C-Means, it was assigned to the cluster with the highest membership value. For Gaussian Mixture Model clustering, it was assigned to the component with the highest posterior probability. For Hierarchical Clustering, it was assigned to the cluster whose centroid was closest. For DBSCAN, which does not natively support out-of-sample assignment, the new sample was assigned to the nearest core point. To ensure that every independent test sample received a prediction, samples outside the epsilon radius were also assigned to the nearest core point rather than being excluded from the accuracy statistics. For deep clustering algorithms (DEC, AEC, DC, DCN, and JULE), the trained encoder was used to extract the low-dimensional embedding of the new sample, and the sample was then assigned to the nearest cluster center in the embedding space. These assignment rules ensure that the independent test samples are processed in a fully out-of-sample manner.

To avoid the statistical bias caused by averaging R^2^ and RMSE across clusters of unequal size, all predictions from the independent test samples were pooled to calculate global R^2^ and RMSE values. Specifically, for each clustering algorithm, the predicted SOM values of all test samples across all clusters were combined into a single vector, and the global R^2^ and RMSE were computed using this pooled vector. This ensures that each test sample contributes equally to the final metrics, regardless of which cluster it belongs to.

The overall workflow consists of five stages. Stage 1: spectral preprocessing using SG smoothing and SNV. Stage 2: fixed 70/30 split of the complete dataset into training and independent testing subsets using the Kennard–Stone algorithm. Stage 3: clustering of soil samples using ten algorithms, including five traditional clustering algorithms (TCAs) and five deep clustering algorithms (DCAs), performed on the training data only. Stage 4: construction of three modeling schemes: (a) traditional global modeling, (b) soil type-based grouping modeling, and (c) spectral similarity-based grouping modeling, with all models trained on the training data only. Stage 5: evaluation of model performance on the same independent test set using pooled global R^2^ and RMSE, and statistical comparison between the DCA and TCA groups. The three modeling schemes are further detailed in Section 2.8.

### 2.8. Three Modeling Schemes

To systematically evaluate the benefit of spectral similarity-based grouping, three modeling schemes were compared in this study. In all three schemes, the complete dataset was first partitioned into 70% training and 30% independent testing subsets using the Kennard–Stone algorithm. This split was performed once and fixed for all schemes and all clustering algorithms. The independent test set was held out before any standardization, clustering, representation learning, or model development, and was never used to define cluster boundaries or to select hyperparameters.

The first scheme is traditional global modeling. Its objective is to establish a single calibration model using all training samples, regardless of their soil type or spectral group. The main steps are: (1) split the complete dataset into 70% training and 30% independent testing using the Kennard–Stone algorithm; (2) train one PLSR model and one CNN model on the full training set; and (3) predict the SOM content of all independent test samples using the global models. This scheme serves as the baseline and does not involve any grouping.

The second scheme is soil type-based grouping modeling. Its objective is to evaluate whether grouping samples by conventional soil taxonomy improves prediction accuracy. The main steps are: (1) split the complete dataset into 70% training and 30% independent testing using the Kennard–Stone algorithm; (2) group the training samples according to their soil type labels in the CSSL and the Xinjiang dataset; (3) train a separate PLSR and CNN model for each soil type using only the training samples of that soil type; and (4) for each independent test sample, assign it to the corresponding soil type model according to its soil type label and predict its SOM content. This scheme tests the hypothesis that soils of the same taxonomic type have similar spectral–SOM relationships.

The third scheme is spectral similarity-based grouping modeling. Its objective is to evaluate whether grouping samples by spectral similarity, rather than by soil type, produces more homogeneous groups and improves prediction accuracy. The main steps are: (1) split the complete dataset into 70% training and 30% independent testing using the Kennard–Stone algorithm; (2) apply each of the ten clustering algorithms to the standardized training data only; (3) assign each training sample to a spectral cluster; (4) train a separate PLSR and CNN model for each cluster using only the training samples assigned to that cluster; (5) for each independent test sample, assign it to a cluster using the out-of-sample rules described in Section 2.7; and (6) predict its SOM content using the matched cluster-specific model. This scheme is the main contribution of the study.

The three schemes differ in three key aspects. First, the grouping criterion is different: no grouping in the global scheme, soil type in the second scheme, and spectral similarity in the third scheme. Second, the number and specificity of models are different: one global model, one model per soil type, or one model per spectral cluster. Third, the assumption about spectral–SOM relationships is different: the global scheme assumes a single relationship across all samples; the soil type scheme assumes that taxonomic type captures the relevant spectral variation; and the spectral similarity scheme assumes that spectral shape is a better indicator of the local spectral–SOM relationship. These differences are also illustrated in the methodological workflow.

## 3. Results

### 3.1. Clustering Results and Analysis

To determine the optimal number of clusters based on soil spectral similarity, this study assessed the silhouette coefficients of ten clustering algorithms across cluster numbers ranging from 1 to 20 (see Figure 2). The resulting coefficients for all algorithms fell within the interval [0, 1], indicating that soil samples can be effectively grouped according to spectral similarity, with high intra-cluster homogeneity and clear separation between clusters. The optimal cluster numbers for the ten algorithms corresponded to silhouette coefficients between 0.65 and 0.83. According to the interpretive criteria established by Kaufman et al. [59]. These values indicate that the clustering results exhibit good inter-cluster separation and intra-cluster cohesion.

Figure 3 presents a two-dimensional PCA projection of the grouped samples from the Chinese Soil Spectral Library (CSSL) and the local Xinjiang dataset, as clustered by the ten algorithms. The five traditional clustering algorithms (TCAs) identified 5 to 11 spectral groups, while the five deep clustering algorithms (DCAs) produced finer classifications, ranging from 5 to 13 clusters. This divergence suggests that DCA methods more effectively extract complex, embedded information from soil hyperspectral data. By detecting subtle spectral variations, such as minor differences in the depth, width, and symmetry of absorption features related to soil organic matter (SOM) content and composition. DCAs can further partition “similar” soil samples identified by TCAs, resulting in a more refined spectral taxonomy. In the visualizations derived from the ten clustering methods, the dense distribution of sample features within most groups reflects strong intra-cluster similarity and consistent representation in the selected feature space. Moreover, with the exception of the Gaussian Mixture Model Clustering (GMMC), the remaining algorithms produced clusters with only limited gaps between groups, indicating some degree of overlap or proximity in the feature space. This pattern confirms that the clustering methods successfully identified meaningful spectral distinctions among different soil samples.

Furthermore, during the clustering analysis process, we found that there were distinct spectral shape differences between different clusters in the visible-light-near-infrared wavelength range, which had clear mineralogical and organic matter-indicative significance. Taking the main clusters obtained by the autoencoder clustering (AEC) method as an example, after extracting the average spectral curves of the samples within each cluster and calculating the first-order derivatives, Cluster 1 exhibited lower reflectance near 550 nm and 650 nm, accompanied by a weak absorption shoulder peak. This corresponded to the overall darkening effect caused by high organic matter content in the soil and the electronic transition absorption of iron ions (Fe^3+^). At the same time, this cluster showed obvious hydration hydroxyl absorption peaks near 1400 nm, 1900 nm, and 2200 nm, especially at 2200 nm, where the absorption depth and symmetry were good, indicating that it contained more layered silicate clay minerals (such as montmorillonite or illite). Cluster 2 exhibited a broad and gentle absorption feature in the near-infrared short-wave region (1100–1300 nm), which was usually related to the double-frequency vibration of Al-OH groups in kaolinite, and the asymmetry of the 2200 nm absorption peak further supported the existence of kaolinite. In contrast, Cluster 3 had a generally higher reflectance in the visible light band and weaker absorption features, but a sharp absorption peak valley appeared near 2300 nm, which corresponded to the bending and stretching vibration combination frequency of C-O bonds in carbonate minerals (calcite, dolomite), indicating that this part of the soil samples might be developed in calcareous parent rock or arid semi-arid environments. For the spherical distribution clusters formed by deep clustering algorithms (DCAs, such as DEC and DCN), the spectral center of the clusters showed a continuous spectral slope in the 2100–2300 nm region instead of independent absorption peaks, suggesting that there was a certain coupling mechanism between organic matter and clay minerals, resulting in smoothed absorption features. It is worth noting that some outlier clusters partitioned by traditional clustering algorithms (TCAs, such as K-Means and DBSCAN) showed abnormal reflection peaks in the 400–500 nm region, which was often caused by the uneven distribution of iron and manganese nodules or iron oxide films on the surface, leading to enhanced surface scattering. The deep clustering algorithms, due to the nonlinear filtering in the low-dimensional embedding space, can effectively suppress such local noise, making the spectra within the clusters more focused on stable absorption features closely related to the soil formation process.

### 3.2. Similarity of Spectral Features of Different Soil Clusters

To assess the inherent consistency of grouping results from different clustering algorithms, this study reduced the soil sample clusters obtained from 10 algorithms to a two-dimensional space (PC1, PC2, and PC3) using principal component analysis (PCA) and conducted similarity tests (results shown in Figure 4). All ten clustering methods demonstrated reasonable to moderately good separation between soil groups, reflecting distinguishable differences in the spectral signatures representing distinct soil types. Except for Gaussian mixture modeling (GMM) and K-Means, the PC1, PC2, and PC3 components of the clustering results from the remaining eight algorithms exhibited significant overlap in the 0–2.5 and 2.5–5.0 ranges. This indicates that despite differing algorithmic principles, these methods exhibit significant consistency in recognizing spectral patterns of soil samples within this feature space region. Such consistency suggests that spectral characteristics in this region exhibit a significant common response to soil organic matter (SOM). In contrast, the GMM and K-Means clustering results exhibit more dispersed distribution patterns in the same region across the PC2 and PC3 dimensions. This suggests that the probabilistic model characteristics of these two algorithms may make them more sensitive to continuous gradients or mixed states in soil spectra, leading to distinct grouping patterns. In-depth analysis of each clustering algorithm revealed that soil groups classified by the Autoencoder (AEC) clustering method exhibited overlap across the three principal component feature dimensions. Simultaneously, most groups formed classification clusters with clearer boundaries and greater internal cohesion in the PCA space. This indicates that the AEC method better identifies spectral feature differences between distinct soil samples and holds an advantage in capturing and separating core spectral feature patterns driven by varying SOM levels.

Notably, we found that the Deep Clustering Algorithm (DCA) tends to form clear hyperspherical distributions in PCA space for soil spectral clustering results, whereas the Traditional Clustering Algorithm (TCA) is more prone to generating arbitrarily shaped distributions. This finding indicates that TCAs are susceptible to noise, redundant bands, and complex nonlinear relationships within soil spectra. In contrast, DCAs more effectively extract low-dimensional representations when clustering soil spectral data. The automatically learned feature space better aligns with the Euclidean distance assumption, enabling linear dimensionality reduction methods like PCA to better preserve the clustering structure.

Figure 5 shows the aggregated Vis-NIR spectra of each cluster obtained through AEC. The average spectra of different clusters show significant differences in reflectance levels and absorption characteristics. Clusters 1, 3, 5, and 7 have overall higher reflectance and have clear absorption peaks at approximately 1400, 1900, and 2200 nm; Clusters 2, 4, 6, 8, and 9 have lower reflectance and relatively gentle absorption characteristics. Among them, Cluster 5 has the strongest absorption around 1900 nm, and Cluster 9 has the lowest overall reflectance. These spectral morphologies indicate that clustering based on spectral similarity can effectively distinguish soil samples with different spectral response characteristics, providing a clear spectral basis for subsequent component modeling.

### 3.3. Modeling Results

To determine whether the performance differences between deep clustering algorithms (DCAs) and traditional clustering algorithms (TCAs) were statistically significant, we conducted non-parametric tests on the average R^2^ and RMSE values. Because the sample size was small (five DCAs and five TCAs) and the distributions could not be assumed to be normal, the Mann–Whitney U test (also known as the Wilcoxon rank-sum test) was used. A *p*-value of less than 0.05 was considered statistically significant.

To validate the feasibility of the spectral similarity modeling approach proposed in this study, for each clustering algorithm, the clustering model was trained on the 70% training set only. The 30% independent test samples were then assigned to the resulting clusters using the out-of-sample rules described in Section 2.7, and each test sample was predicted using the model of the cluster to which it was assigned. The training samples were used to train the PLSR and CNN models, while the independent test samples were employed to evaluate model accuracy. The statistical averages of the predictive performance of each clustering algorithm’s sample group within the independent test set are shown in Figure 6. The average R^2^ and RMSE ranges for the PLSR and CNN models established from the 10 clustered datasets were 0.63–0.85 and 1.07–3.55 g kg^−1^, respectively. Specifically, the PLSR and CNN models based on deep clustering (DCA) achieved average R^2^ and RMSE values of 0.76, 0.80, 1.97 g kg^−1^, and 1.62 g kg^−1^, respectively. In contrast, models based on traditional clustering (TCA) yielded R^2^ and RMSE values of 0.70, 0.75, 2.56 g kg^−1^, and 2.05 g kg^−1^, respectively. Models developed using the DCA approach demonstrated an R^2^ improvement of 1.33–14.29% and an RMSE reduction of 23.05–44.90% compared to those based on TCA. These results indicate that among modeling approaches based on spectral similarity, DCAs more effectively extract soil spectral information, thereby delineating more representative sample groups. Furthermore, among the 10 clustering algorithms tested, the Autoencoder Clustering (AEC) algorithm demonstrated the best performance. PLSR and CNN models developed using soil groups partitioned by AEC exhibited optimal predictive capabilities.

To further explain why certain clusters yielded better PLSR and CNN performance, we examined the relationship between cluster membership and the main spectral absorption features. The AEC-derived clusters, which achieved the highest prediction accuracy, showed clear separation in the visible–near-infrared region. Clusters with lower reflectance at 550–650 nm and pronounced absorption at 1400, 1900, and 2200 nm were associated with higher SOM content and clay minerals. In contrast, clusters with high visible reflectance and a sharp absorption feature near 2300 nm were associated with carbonate-rich soils. These spectrally distinct groups reduced the confounding effects of mineralogy, soil texture, moisture, and iron oxides on SOM prediction. As a result, the SOM-spectra relationship within each AEC cluster was more stable, and the PLSR and CNN models trained on these clusters achieved higher R^2^ and lower RMSE. Traditional clustering algorithms, which rely on linear distance metrics, often mixed samples with different mineralogical backgrounds and interference levels, leading to noisier training data and poorer prediction performance.

The Mann–Whitney U test showed that the DCA group achieved significantly higher average R^2^ than the TCA group (*p* = 0.032). The DCA group also achieved significantly lower average RMSE than the TCA group (*p* = 0.028). In addition, the AEC-based models produced the highest R^2^ and the lowest RMSE among all ten clustering algorithms, and the difference between AEC and the other algorithms was statistically significant (*p* < 0.05). These results confirm that the superior performance of DCA, and especially AEC, is not due to random variation but is statistically significant.

We also monitored the training and validation loss curves for the CNN models. In all clusters, the training and validation losses converged to similar values, and no significant gap was observed, indicating that overfitting was not severe. The average difference between training and validation RMSE was less than 0.3 g kg^−1^.

It should be noted that the R^2^ and RMSE values reported here are pooled global metrics, computed by combining the predictions of all test samples across all clusters. This avoids the bias that would arise from simply averaging the metrics of clusters with different sample sizes.

### 3.4. The Prediction Accuracy of the Inversion Model Based on the Two Methods

Table 2 presents the predictive performance of PLSR and CNN models under three distinct modeling approaches. Results indicate that the classification model based on soil spectral similarity achieved the best performance, followed by the soil type classification model, while the traditional modeling approach demonstrated the weakest performance. Among these, the PLSR and CNN models attained the highest prediction accuracy under the spectral similarity modeling approach. Compared with models established using the traditional modeling approach, the spectral similarity-based approach increased R^2^ from 0.65–0.71 to 0.81–0.85 (relative increase: 14.08–30.77%) and reduced RMSE from 2.49–3.24 g kg^−1^ to 1.07–1.39 g kg^−1^ (relative reduction: 44.18–66.98%). Compared with models established by the soil type classification approach, the spectral similarity approach further increased R^2^ from 0.70–0.74 to 0.81–0.85 (relative increase: 9.46–21.43%) and reduced RMSE from 1.91–2.56 g kg^−1^ to 1.07–1.39 g kg^−1^ (relative reduction: 27.23–58.20%). These results indicate that classifying identical soil samples before establishing predictive models enables more effective exploration of intrinsic relationships between spectral data and soil properties, significantly enhancing model prediction accuracy. However, traditional soil taxonomic information in soil-classification-based modeling approaches struggles to fully capture key spectral variation features and dependencies. In contrast, classification methods based on spectral features more effectively capture and represent information relevant to target attributes.

It should be noted that the soil type-based grouping strategy was affected by the uneven sample sizes of different soil types. Some soil types had many more samples than others, which may have led to unstable model training for soil types with fewer samples. In contrast, spectral similarity-based grouping produced clusters of more balanced sizes, which helped improve model stability and prediction accuracy.

### 3.5. Explore Whether the Spatial Distribution Characteristics of Soil Samples Are Correlated with Their Spectral Characteristics

**Figure 7:**
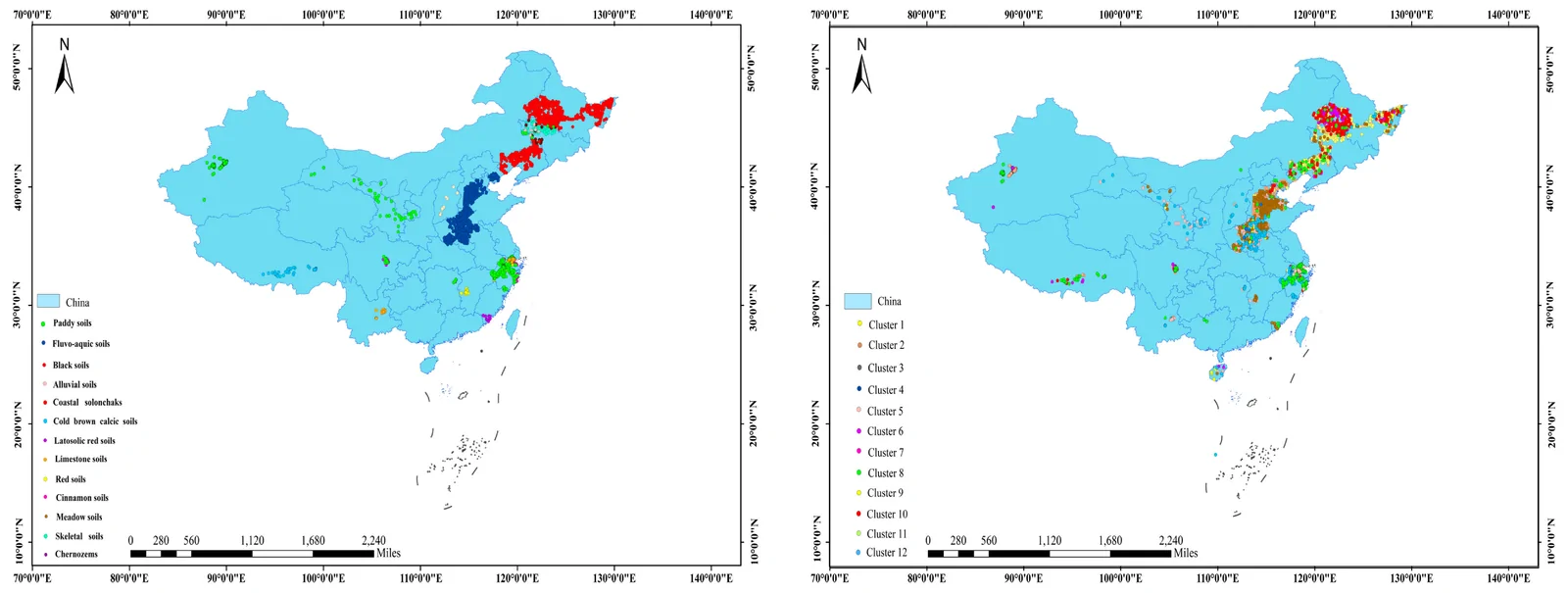
Sample distribution map of the CSSL dataset based on soil type classification and spectral similarity classification.

By grouping the Chinese Soil Spectral Library (CSSL) and local Xinjiang samples based on soil spectral similarity using an autoencoder (AEC), 12 clusters were obtained. Subsequently, a distribution map of CSSL soil samples was plotted according to 14 soil types and 12 clusters, as shown in Figure 7. Among them, the traditional modeling approach refers to using all the data to establish a global model. Integrating the findings from Section 3.2, we observed significant spectral differences among samples of the same soil type. Samples of the same soil type were assigned to different spectral clusters. For example, black soil samples were dispersed across five spectral clusters, exhibiting visible–near-infrared reflectance differences ranging from 12% to 34% (particularly in the 600–800 nm range). This indicates that traditional soil classification systems cannot fully characterize spectral response characteristics. Secondly, soil samples with similar spatial distribution do not necessarily exhibit identical spectral characteristics. Only 44% of samples separated by <50 km belonged to the same spectral cluster, whereas 31% of samples separated by 1000 km showed spectral similarity (Euclidean distance < 0.15). For instance, soils classified as Cinnamon (approximately 115° E, 33° N) in the warm-temperate zone of North China exhibit spectral characteristics highly similar to those of Latosolic red soils (approximately 120° E, 28° N) exhibit high spectral similarity. Their spectral curves show highly consistent absorption troughs at 1350–1450 nm. In contrast, significant spectral differences exist among Northeast black soil samples from spatially proximate locations (approximately 124–133° E).

To quantitatively verify the conclusion that ‘there are significant differences in spectral characteristics within the same soil type’, we further conducted an analysis of spectral dissimilarity within and between soil types. Firstly, for the 14 soil types in the Chinese Soil Spectral Library (CSSL), we calculated the average Euclidean distance between all sample pairs within each soil type, using the standardized spectral reflectance vectors as the intra-type spectral dissimilarity indicator. At the same time, we calculated the average Euclidean distance between sample pairs of different soil types as the inter-type spectral dissimilarity indicator. The results showed that the average intra-type distance was 0.312 ± 0.087, and the average inter-type distance was 0.378 ± 0.102. Although there was some overlap, the independent-samples t-test showed that the differences were extremely significant. What is more noteworthy is that for multiple soil types (such as black soil, brown soil, and wet soil), the intra-type dissimilarity even exceeded the inter-type dissimilarity of some soil types. For example, the average Euclidean distance between samples within black soil was as high as 0.367, while the inter-type distance between black soil and dark brown soil was only 0.284. This indicates that the spectral variation within black soil is greater than the difference between it and another soil type. The above statistical evidence clearly supports that there is no simple one-to-one correspondence between soil type labels and spectral characteristics. Samples of the same soil type can present a degree of dispersion in the spectral feature space that is close to or even exceeds the inter-type difference. This phenomenon may be due to the fact that traditional soil classification mainly relies on soil-forming factors such as parent material, climate, biology, topography, and time, as well as the resulting profile morphological characteristics, while Vis-NIR spectroscopy mainly responds to the chemical composition and physical structure of organic matter, iron oxides, clay minerals, and carbonates in the soil. The dimensions of the soil attributes captured by the two are fundamentally different. Therefore, the spectral characteristics of the same soil type may undergo significant fluctuations due to local changes in soil-forming factors.

**Figure 8:**
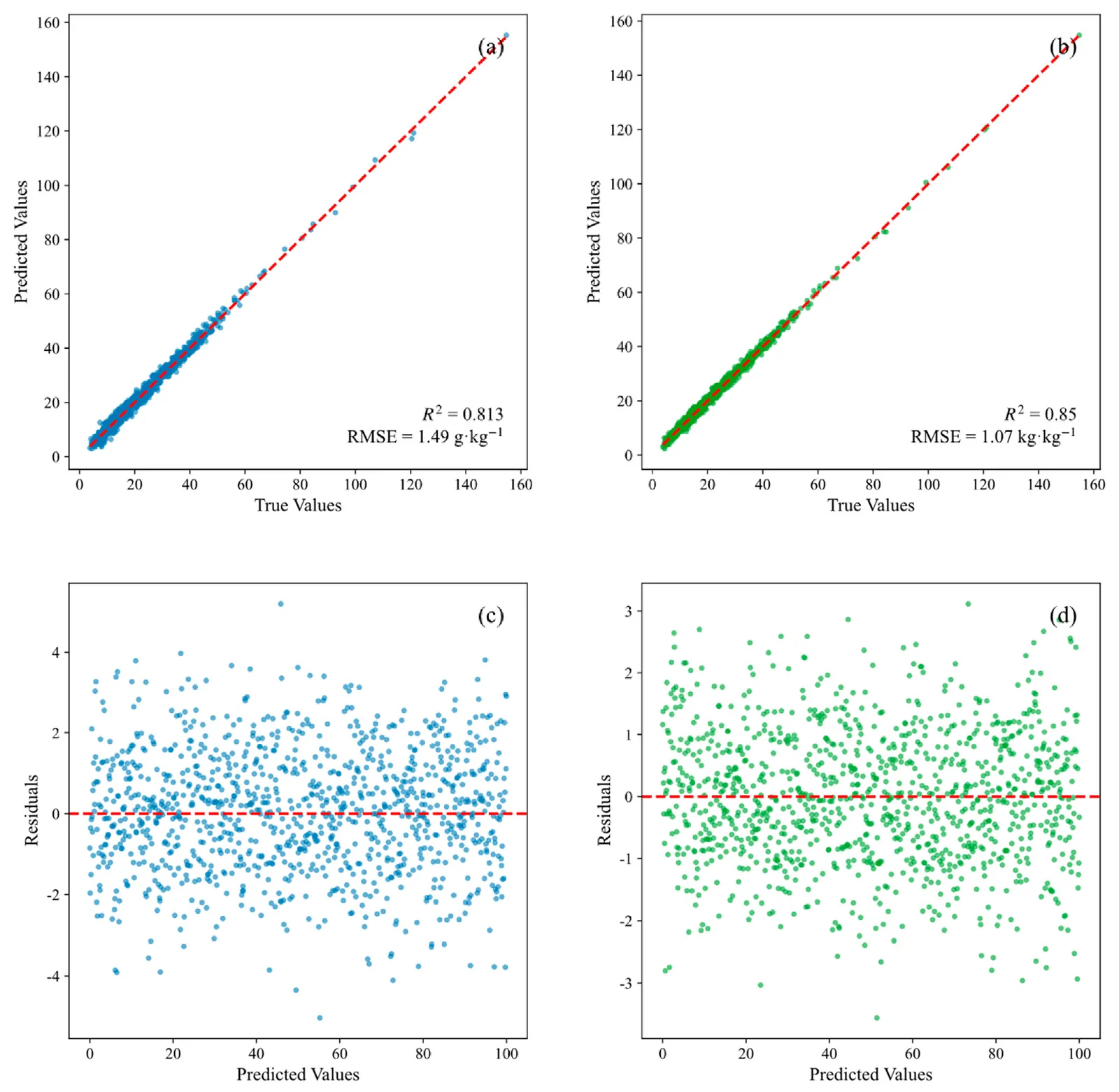
After the test set data was classified by the AEC model, the scatter plots and residual plots of the predicted values of each group compared with the actual values were presented. For a more intuitive display, we plotted the predicted values of each group in a unified dataset. (**a**,**c**) represent the PLSR prediction results, while (**b**,**d**) represent the CNN prediction results. True values means Observed SOC (g/kg), and Predicted values means Predicted SOC (g/kg).

To further assess whether the prediction errors have a systematic correlation with the SOM concentration, independent test samples were assigned to the clusters derived from AEC, and the corresponding group-level models were used for prediction. Figure 8 shows the observed values, predicted values, and corresponding residuals in the aggregated test set. Figure 8a,c display the results of PLSR, while Figure 8b,d show the results of CNN. The scatter plots indicate that both models generally follow a 1:1 line, with CNN clustering more closely around this line. The residual plots show that most residuals are distributed around the zero line, and no significant systematic trend is observed within the predicted SOM range. No cases of severe underestimation in the high SOM region or severe overestimation in the low SOM region were observed. Although there were still a few outliers, their impact was limited. Overall, the clustering method based on AEC did not introduce significant concentration-dependent bias, which supports the reliability of the aggregated R^2^ and RMSE values and confirms that the observed improvement in prediction accuracy was not at the cost of introducing systematic errors.

## 4. Discussion

### 4.1. Deep Clustering Is Superior to Traditional Clustering

When applying the cluster analysis algorithm (TCA) to analyze soil spectral data, the core principle of TCA involves grouping similar data points into clusters by measuring distances between data points [19,22]. However, the complexity of soil spectroscopy stems from its multi-component coupled responses. Signals such as oxygenated functional groups (-OH, C=O) in soil organic matter (SOM), lattice vibrations of clay minerals, charge transfer in iron oxides, and second harmonic absorption of water molecules highly overlap at specific wavelengths (1400 nm, 1900 nm, and 2200 nm) [64]. TCA methods typically rely on simple metrics in feature space, often struggling to decouple these intertwined spectral effects caused by soil properties, resulting in an inability to effectively capture complex spectral data structures [23,64]. In contrast, deep clustering algorithms (DCAs) combine the feature extraction capabilities of deep learning (DL) to more accurately process and learn high-dimensional data. These algorithms typically learn more abstract feature representations that not only effectively reduce dimensionality but also extract more discriminative features, thereby enhancing clustering performance [25]. Furthermore, when establishing regression models for each sample group, it was found that models trained on groups partitioned by DCA generally exhibited higher predictive accuracy. This likely stems from the greater internal feature consistency within DCA-generated groups, enabling better model fitting for these homogeneous samples [6,20]. In contrast, TCAs may combine samples of different types, causing interference during model training and thereby affecting prediction accuracy [13]. Furthermore, the feature representations learned by DCA better reflect the association between soil properties and spectral characteristics. Consequently, when used as input variables for regression analysis, they enable regression models to more effectively capture the relationship between soil properties and spectral features.

We acknowledge that high-dimensional spectral data can cause distance concentration, which may negatively affect distance-based traditional clustering algorithms such as K-Means and Hierarchical Clustering. In this study, the traditional clustering algorithms were applied to the full-spectrum data without external dimensionality reduction, while the deep clustering algorithms learned low-dimensional representations internally. This difference is an inherent characteristic of the two families of methods rather than an intentional bias. Nevertheless, it is possible that applying dimensionality reduction before traditional clustering could improve their performance. Future work could systematically evaluate the effect of PCA, UMAP, or wavelet-based dimensionality reduction on traditional clustering algorithms for soil spectral data. Our main conclusion-that deep clustering algorithms, especially AEC, produce more spectrally homogeneous groups and yield better prediction accuracy-is based on the same input data and the same evaluation framework for all methods, and we believe it remains valid.

We acknowledge that splitting the dataset into clusters reduces the sample size available for each local model. Although we applied regularization and early stopping, small clusters may still lead to less stable CNN training. Future work could address this by collecting more samples for underrepresented clusters or by using transfer learning from a global model.

Our findings are consistent with previous soil spectral-grouping studies in several respects, but they also extend them in important ways. Ward et al. [20] applied spectral clustering to the LUCAS soil database and reported that grouping samples by spectral similarity improved soil organic carbon prediction compared with global modeling. Our results agree with this general conclusion: spectral similarity-based grouping outperformed traditional global modeling for SOM prediction. However, unlike Ward et al. [20], who used a single clustering approach, we systematically compared five traditional and five deep clustering algorithms. This allowed us to show that the choice of clustering algorithm matters, and that deep clustering algorithms, especially AEC, produced more homogeneous groups in our dataset. Similarly, Ramirez-Lopez et al. [18] and Nocita et al. [10] demonstrated the benefits of local modeling using spectrally selected neighbors. Our study differs in that we perform a one-time global partitioning of the entire spectral library into groups, rather than dynamically selecting neighbors for each test sample. This group-level approach produces a fixed set of interpretable spectral groups and enables direct comparison of clustering algorithms. These comparisons help position our contribution relative to existing local modeling and spectral grouping research.

### 4.2. Automatic Encoder Clustering Performed Best in This Study

In visible–near-infrared (Vis-NIR) spectral clustering analysis of soil, the autoencoder clustering (AEC) algorithm demonstrated optimal clustering accuracy and highest modeling precision due to its unique feature extraction capabilities and data processing advantages. This likely stems from AEC’s ability to compress input data into low-dimensional representations (encoded) before decoding them back to original data [46]. This decomposition approach enables more efficient learning of effective representations of the input data, capturing the primary characteristics of the data [47]. Additionally, AEC possesses robust nonlinear mapping capabilities, allowing it to learn complex nonlinear relationships within the data [65]. In soil spectral data, complex nonlinear relationships often exist between spectral characteristics and physical/chemical properties [8,66]. AEC effectively captures these relationships, enabling superior identification of distinct soil sample groups during clustering. Simultaneously, AEC effectively extracts important high-dimensional spectral data and transforms it into low-dimensional features. This not only reduces data redundancy and noise but also allows clustering algorithms to focus on the most useful information for clustering, thereby improving clustering accuracy [67,68]. Furthermore, during training, AEC strives to minimize the discrepancy between input and reconstructed data [68]. This process ensures that learned features retain critical information from the data, making the resulting low-dimensional representation more suitable for subsequent clustering analysis.

Notably, the spectral reflectance characteristics of soil are directly influenced by its physical and chemical properties [9,23]. For instance, organic matter, mineral composition, and moisture content in soil all induce specific changes in spectral characteristics (e.g., shifts in absorption peaks). By learning these features, AEC enables effective separation of spectral information from different soil samples within the feature space, capturing subtle distinctions between them. This allows similar soil types to be grouped together during clustering, significantly enhancing the predictive capability of subsequent regression models for soil properties.

It is important to emphasize that the superior performance of AEC is not merely a statistical artifact. Soil organic matter prediction depends on specific spectral absorption features, including OH and CH absorption near 1400 nm, OH absorption near 1900 nm, and Al-OH and COOH absorption near 2200 nm, as well as overall darkening in the visible region [20]. AEC clusters that showed strong absorption at these wavelengths and lower visible reflectance likely represent soils with high SOM and clay content. In contrast, clusters dominated by carbonate absorption at 2300 nm or iron oxide features below 600 nm may have lower SOM or different mineralogy. By separating these spectrally distinct groups, AEC reduces the confounding effects of mineralogy, texture, and moisture on SOM prediction [68]. This is consistent with previous studies showing that local models based on spectral similarity outperform global models when soil heterogeneity is high. The clustering results show that AEC produced groups with higher internal spectral homogeneity and better SOM prediction accuracy than the other algorithms in our dataset. However, we would like to emphasize that these results demonstrate an association, not a causal mechanism. The observed spectral differences among clusters, such as lower reflectance at 550–650 nm and stronger absorption at 1400, 1900, and 2200 nm, are consistent with variations in SOM, clay minerals, and iron oxides, but we did not measure these properties directly. Therefore, the mineralogical and organic interpretations should be regarded as plausible explanations rather than proven mechanisms. Similarly, the suggestion that deep clustering suppresses noise more effectively than traditional clustering is based on the observed clustering patterns and model performance, but it is not a direct measurement of noise suppression. Future work with independent mineralogical and textural data could test these hypotheses more rigorously.

It is worth explaining mechanistically why the simpler AEC model outperformed more complex end-to-end architectures such as DEC and DCN in this study. DEC and DCN jointly optimize reconstruction and clustering losses in a single training process. Although this end-to-end design is powerful in principle, it introduces a trade-off between two objectives that are not always aligned [68]. The clustering loss can push the latent representation to form compact clusters, while the reconstruction loss tries to preserve all spectral information, including noise and irrelevant variation. In soil Vis-NIR data, the relevant SOM signal is weak and overlaps with mineral, moisture, and iron oxide features. When the clustering loss dominates, the encoder may discard spectral details that are useful for SOM prediction; when the reconstruction loss dominates, the latent space may not be sufficiently discriminative for clustering [69]. This trade-off is difficult to balance, especially with limited sample sizes per cluster.

In contrast, AEC adopts a two-stage strategy: it first trains an autoencoder purely for reconstruction, and then performs K-Means clustering in the learned embedding space. This decouples representation learning from clustering [70]. The autoencoder is encouraged to preserve the overall spectral shape and the main absorption features, while K-Means groups samples according to their similarity in the embedding space. Because the two objectives are optimized separately, AEC avoids the instability caused by conflicting losses. This is particularly advantageous for soil spectral data, where the number of samples is moderate, and the spectral variation is complex. The simpler and more stable training procedure of AEC also reduces the risk of overfitting and produces more consistent clusters across different random initializations. These factors explain why AEC achieved the best clustering accuracy and the highest prediction performance in our study [71].

It is important to note that the superior performance of AEC was observed in this specific dataset and for SOM prediction. We do not claim that AEC is universally superior to other clustering algorithms. Different datasets, soil properties, or spectral ranges may favor different clustering approaches. The relative performance of clustering algorithms depends on the data characteristics, and our results should be interpreted within the scope of this study. Future work should evaluate these algorithms on other soil spectral libraries and target properties to test the generality of our findings.

### 4.3. The Idea of Classification Modeling Based on Spectral Feature Similarity Is Superior to Soil Classification Modeling

Modeling based on soil types may sometimes result in insufficient prediction accuracy. This is because traditional soil classification systems are typically based on broad criteria (such as pedogenic conditions and processes), which may fail to adequately capture subtle spectral variations [15]. Consequently, models train on all samples within the same soil type as homogeneous, establishing a generalized model. This approach overlooks the variability in spectral characteristics among samples within the same soil type and their subtle correlations with the inversion target (e.g., soil organic matter). This makes it difficult for the model to capture key features, thereby affecting prediction accuracy [13,69].

Furthermore, modeling based on soil types lacks flexibility for different subclasses, potentially hindering the model’s adaptability to variations in characteristics and soil properties [69]. This can degrade prediction performance for certain soil types, particularly regarding marginal or atypical samples. In contrast, clustering (TCA) groups spectrally similar samples into the same category, making samples within each soil group more homogeneous in characteristics. This enhances the model’s ability to capture features and identify more regular correlations between soil properties and spectral data. This homogeneity enables models trained for each soil group to more accurately describe the intrinsic characteristics of samples within that group [20,23]. Each soil group can be trained with an independent model tailored to its specific characteristics. This approach allows models to better accommodate differences between categories, thereby improving overall predictive capability. Furthermore, post-clustering models can capture subtle variations and features between samples with greater precision, subtleties often overlooked by traditional classification methods [15]. Furthermore, models constructed with small sample sizes and high inter-sample variability are prone to overfitting [9]. Cluster-based modeling mitigates this risk by leveraging more homogeneous sample sets, thereby enhancing model generalization. In contrast to soil type classification, spectral similarity clustering captures continuous variations in mineral composition, texture, moisture status, and iron oxide content, which are directly expressed in the spectral response [70,71]. This explains why spectral clustering produced more homogeneous groups and better prediction accuracy than soil type grouping.

In summary, modeling based on predefined soil types faces limitations when handling complex and heterogeneous soil data. By performing spectral clustering followed by modeling for each soil fraction, the approach better accommodates inter-sample variations, yielding higher accuracy and robustness. This method not only enhances modeling effectiveness but also deepens our understanding of the relationship between soil properties and spectral characteristics.

### 4.4. The Spectral Similarity and Spatial Similarity of Soil

Cluster analysis of soil visible–near-infrared (Vis-NIR) spectra revealed that spatially adjacent samples did not necessarily exhibit similar spectral characteristics, while significant spectral variations were also observed among samples of the same soil type. We hypothesize that this phenomenon primarily stems from the complexity of soil formation processes, which are influenced by a combination of factors including climate, topography, parent material, vegetation, and human activities [72,73]. Even in spatially adjacent areas, soil formation processes and composition may differ [74]. For instance, different types of parent rock may exist within the same region, influencing soil mineral composition and properties. Furthermore, high spatial heterogeneity can also affect soil spectral characteristics [15,74]. This heterogeneity may stem from climate variations, soil fertilization, cultivation practices, or natural factors such as flooding and soil erosion. These factors can cause adjacent soil samples to exhibit differences in spectral reflectance and absorptance, thereby affecting their spectral similarity. Secondly, soil spectral data typically exhibit high dimensionality and complexity. Different soil types may show significant variations in light reflection and absorption characteristics due to their material composition (e.g., organic matter, minerals, clay content) [75]. Even spatially proximate samples may exhibit distinct spectral responses due to differing compositions and structures. This nonlinear response means spectral similarity between adjacent samples is not always consistent. Moreover, fundamental differences in composition and structure between soil types manifest spectrally as distinct absorption peaks and reflectance characteristics, resulting in markedly varying spectral similarity among different soil types [13,76].

In summary, spatially proximate soil samples do not necessarily exhibit similar spectral similarity. Even among samples of the same soil type, significant variations in spectral similarity exist. This phenomenon is primarily attributed to the complexity of soil formation processes, spatial heterogeneity at short distances, nonlinear spectral responses, and inherent differences between soil types.

### 4.5. Limitations and Future Work

We also acknowledge two important limitations. First, splitting the dataset into clusters reduced the sample size available for training each local model, and some clusters were relatively small [68]. Although we applied regularization, early stopping, and data augmentation, small clusters may still lead to less stable CNN training and higher variance in performance. Future work could address this by collecting more samples for underrepresented clusters or by using transfer learning from a global model. Second, our validation was not geographically independent [70].

## 5. Conclusions

This study addresses the issue of spectral feature variations caused by sample heterogeneity in the field of soil hyperspectral analysis by innovatively proposing a solution based on spectral similarity grouping. Using the China Soil Spectral Library (CSSL) and local Xinjiang samples as data sources, the following conclusions were drawn: (1) Compared with traditional global modeling, spectral similarity-based grouping increased R^2^ from 0.65–0.71 to 0.81–0.85 and reduced RMSE from 2.49–3.24 g kg^−1^ to 1.07–1.39 g kg^−1^. (2) Compared to traditional soil classification modeling, this approach substantially enhances the accuracy of SOM prediction models, with R^2^ increasing from 0.70–0.74 to 0.81–0.85 (relative increase: 9.46–21.43%) and RMSE decreasing from 1.91–2.56 g kg^−1^ to 1.07–1.39 g kg^−1^ (relative reduction: 27.23–58.20%). (3) Deep Clustering Algorithm (DCA) more accurately identifies and aggregates soil samples with highly similar spectral features than the Traditional Clustering Algorithm (TCA). Among 10 clustering methods, AEC demonstrated the highest clustering accuracy and modeling performance; (4) In the evaluated dataset, spatial proximity was not consistently associated with spectral similarity. Some spatially adjacent samples belonged to different spectral clusters, while some distant samples were assigned to the same cluster. These observations suggest that soil type labels alone were insufficient to capture spectral variation relevant to SOM prediction in this dataset. However, further analysis is needed to quantify the relationship between spatial distance and spectral similarity.

These findings are based on the Chinese Soil Spectral Library and the Xinjiang dataset and are specific to SOM prediction. They should be validated in other regions, soil spectral libraries, and for other soil properties before broad application. The validation strategy used in this study evaluated the relative ability of different grouping strategies to produce internally homogeneous clusters. It did not evaluate the full end-to-end workflow with a single independent test set held out before clustering. Future work should adopt a global hold-out test set to evaluate the operational performance of the proposed approach. In addition, some clusters were relatively small, which may have led to less stable CNN training. Future work could address this by collecting more samples for underrepresented clusters or by using transfer learning. Geographically independent validation is also needed before claiming regional transferability.

## Figures and Tables

**Table 1:** Descriptive statistics of soil organic matter (SOM) content (g kg^−1^) for each soil group in the Chinese Soil Spectral Library (CSSL) and the Xinjiang dataset. Soil groups follow the [Chinese Soil Taxonomy] classification. Note: Samples = number of soil samples; Min = minimum; Max = maximum; Mean = mean value; SD = standard deviation. All statistical values refer to SOM content in g kg^−1^.

Soil Group	Samples	Min	Max	Mean	SD
Meadow soils	60	16.24	50.23	35.39	7.89
Paddy soils	810	3.74	61.5	28.3	10.2
Black soils	399	4.81	94.6	35.34	12.02
Chernozems	180	4.31	46.51	13.24	5.8
Purplish soils	261	1.57	50.1	29.98	9.54
Cinnamon soils	208	5.53	35.1	14.2	5
Cold brown calcic soils	89	1.67	84.94	44.77	36
Red soils	172	3.39	59.3	18.40	10.44
Limestone soils	315	4.35	78	18.36	11.4
Latosolic red soils	325	3.2	67.1	15	6.8
Skeletal soils	314	4.1	58.8	25.06	11.66
Fluvo-aquic soils	397	3.95	60.5	19.23	10.94
Coastal solonchaks	114	3.71	31.5	12.46	10.94
Total	3644	1.57	94.6	45.07	14.2

**Table 2:** Average performance statistics table of the three modeling schemes.

Spectral Modeling Scheme	PLSR		CNN	
	*R* ^2^	*RMSE* (g kg^−1^)	*R* ^2^	*RMSE* (g kg^−1^)
Spectral similarity	0.81	1.39	0.85	1.07
Soil type	0.70	2.56	0.74	1.91
Traditional modeling	0.65	3.24	0.71	2.49

## Data Availability

The authors do not have permission to share data.

## Supplementary Materials

The following supporting information can be downloaded at https://www.mdpi.com/article/10.3390/s26216824/s1; Figure S1: The structure diagram of K-Means clustering algorithm; Figure S2: The structure diagram of Fuzzy C-means clustering algorithm; Figure S3: The structure diagram of DBSCAN clustering algorithm; Figure S4: The structure diagram of Gaussian mixture model clustering algorithm; Figure S5: The structure diagram of Hierarchical clustering algorithm; Figure S6: The structure diagram of Deep Embedded Clustering algorithm; Figure S7: The structure diagram of Autoencoder clustering algorithm; Figure S8: The structure diagram of Deep Clustering algorithm; Figure S9: The structure diagram of Deep Clustering Network algorithm; Figure S10: The structure diagram of Joint Unsupervised Learning of Deep Representations and Image Clusters algorithm.

## Abbreviations

The following abbreviations are used in this manuscript:

TCA	Traditional Clustering Algorithms
DCA	Directory of Open Access Journals
AEC	Automatic encoding clustering
SOM	Soil organic matter
ML	Machine learning
DL	Deep learning
SSL	Soil spectral libraries
AI	Artificial intelligence
CSSL	Chinese Soil Spectral Library
SG	Savitzky–Golay
SNV	Standard Normalization Variation
DEC	Deep embedded clustering
DC	Deep clustering
DCN	Deep clustering network
