# Supplementary material for: Spectral Similarity-Based Grouping Improves Laboratory Vis-NIR Prediction of Soil Organic Matter Under Soil Heterogeneity
Source: Sensors (Basel). 2026 Oct 28;26(21):6824. doi: 10.3390/s26216824 (PMC13766184; doi:10.3390/s26216824)
Supplement: Supplementary file 1 [file sensors-26-06824-s001.zip › sensors-4541189-supplementary.pdf]

Spectral similarity-based grouping improves laboratory Vis-NIR prediction of soil organic matter under soil heterogeneity

Jia Deng [a], Junjie Liu [a], Bifeng Hu [b], Jie Peng [a,c,d*], Zhou Shi [e]

[a] College of Agriculture, Tarim University, Alar 843300, China

[b] Key Laboratory of Data Science in Finance and Economics, Jiangxi University of Finance and Economics, Nanchang 330013, China

[c] Key Laboratory of Genetic Improvement and Efficient Production for Specialty Crops in Arid Southern Xinjiang of Xinjiang Corps, Alar 843300, China

[d]Key Laboratory of Tarim Oasis Agriculture(Tarim University), Ministry of EduTCAtion, Alar 84 3300, China

[e] College of Environment and Resource Sciences, Zhejiang University, Hangzhou 310058, China

[*] Corresponding author: Jie Peng (pjzky@163.com)

Supplementary Figures:

Figure S1 The structure diagram of K-Means clustering algorithm.
Figure S2 The structure diagram of Fuzzy C-means clustering algorithm.
Figure S3 The structure diagram of DBSCAN clustering algorithm.
Figure S4 The structure diagram of Gaussian mixture model clustering algorithm.
Figure S5 The structure diagram of Hierarchical clustering algorithm.
Figure S6 The structure diagram of Deep Embedded Clustering algorithm.
Figure S7 The structure diagram of Autoencoder clustering algorithm.
Figure S8 The structure diagram of Deep Clustering algorithm.
Figure S9 The structure diagram of Deep Clustering Network algorithm.
Figure S10 The structure diagram of Joint Unsupervised Learning of Deep Representations and Image Clusters algorithm.

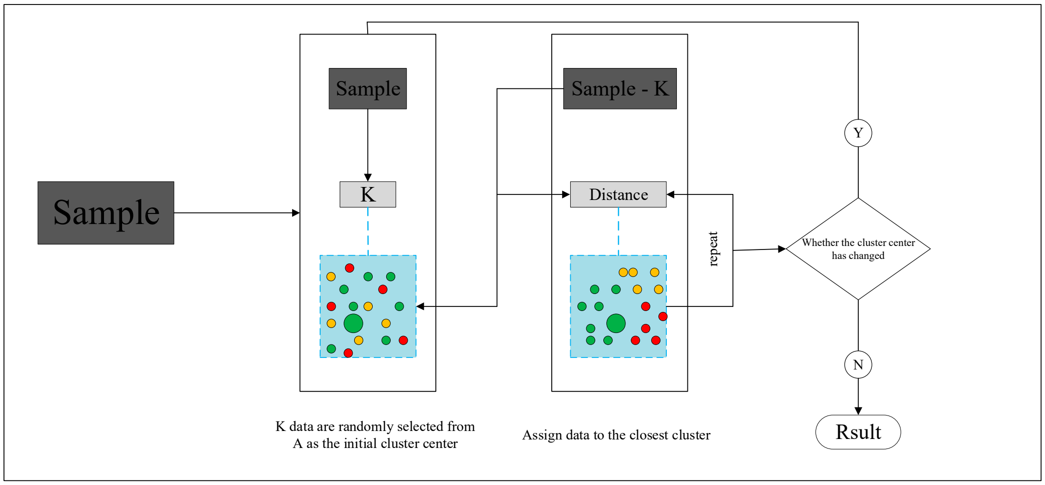

Figure S1 The structure diagram of K-Means clustering algorithm.

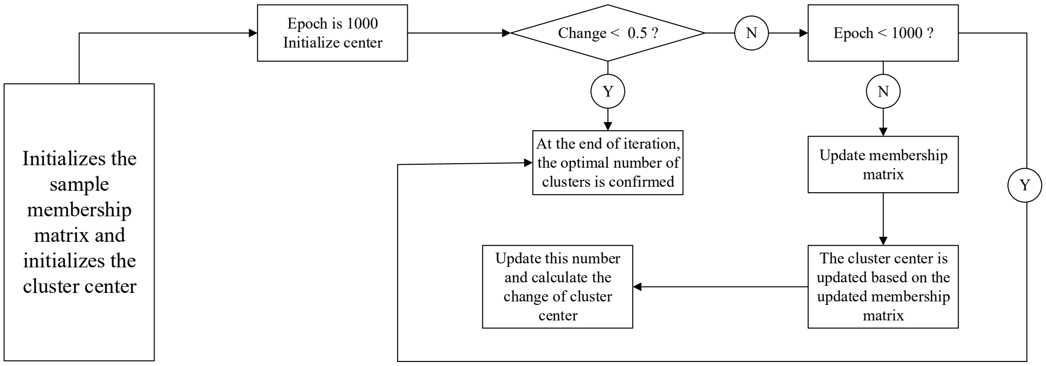

Figure S2 The structure diagram of Fuzzy C-means clustering algorithm.

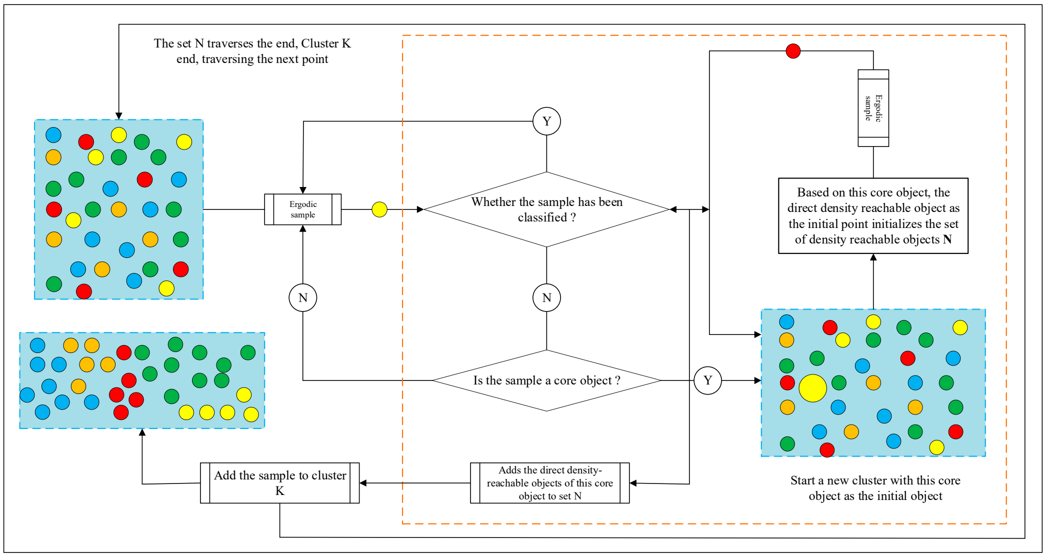

Figure S3 The structure diagram of DBSCAN clustering algorithm.

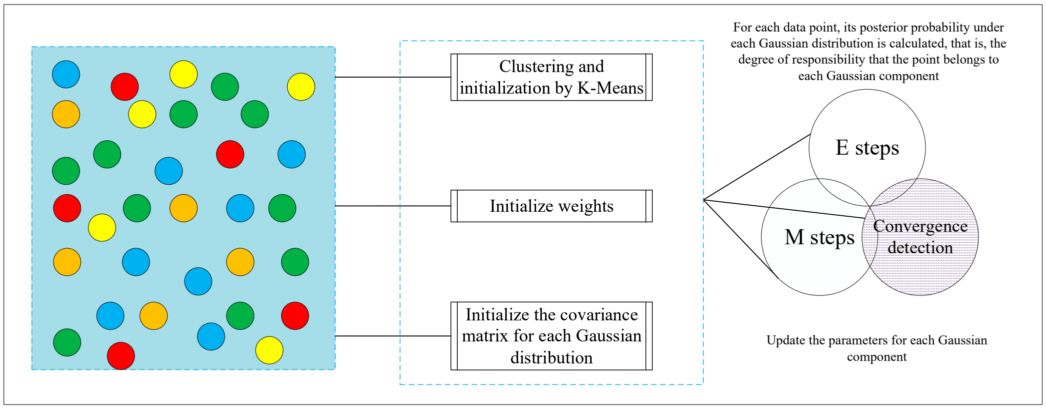

Figure S4 The structure diagram of Gaussian mixture model clustering algorithm.

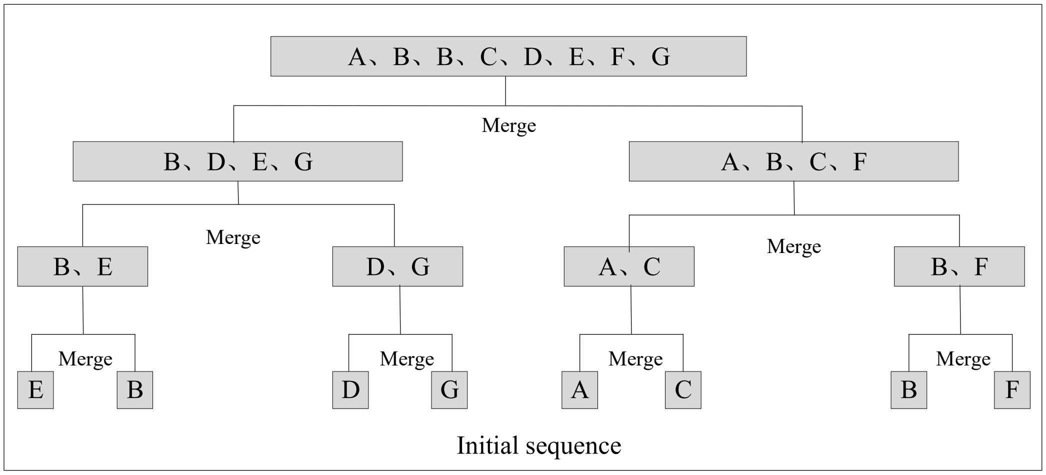

Figure S5 The structure diagram of Hierarchical clustering algorithm.

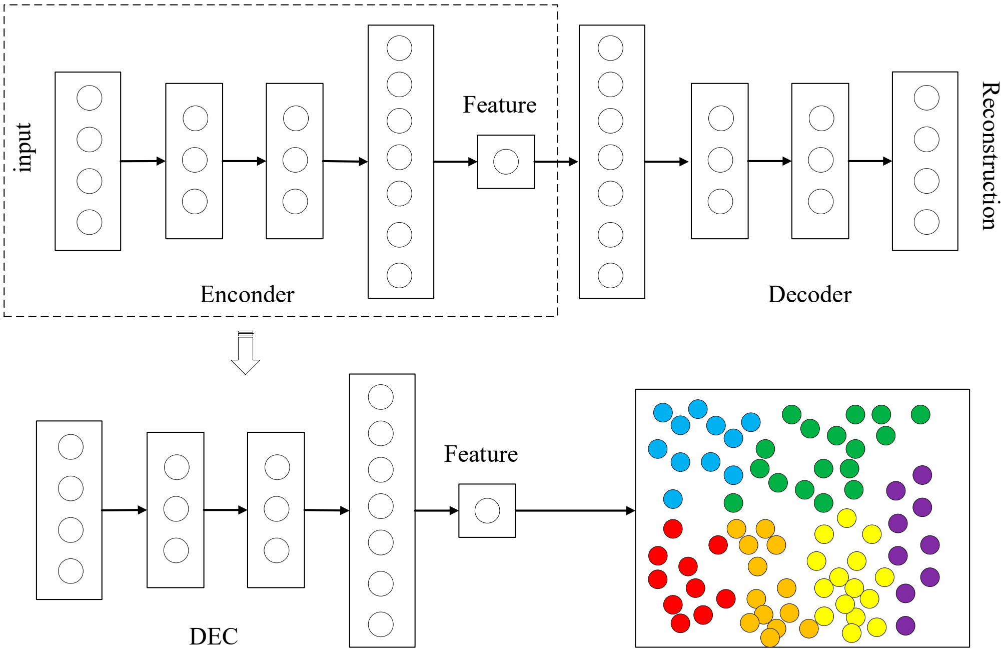

Figure S6 The structure diagram of Deep Embedded Clustering algorithm.

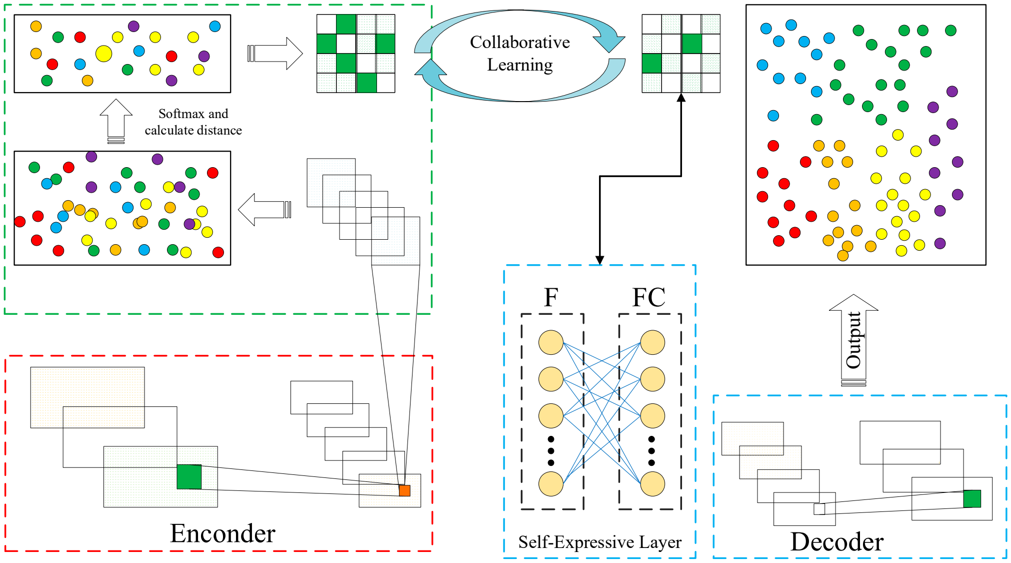

Figure S7 The structure diagram of Autoencoder clustering algorithm.

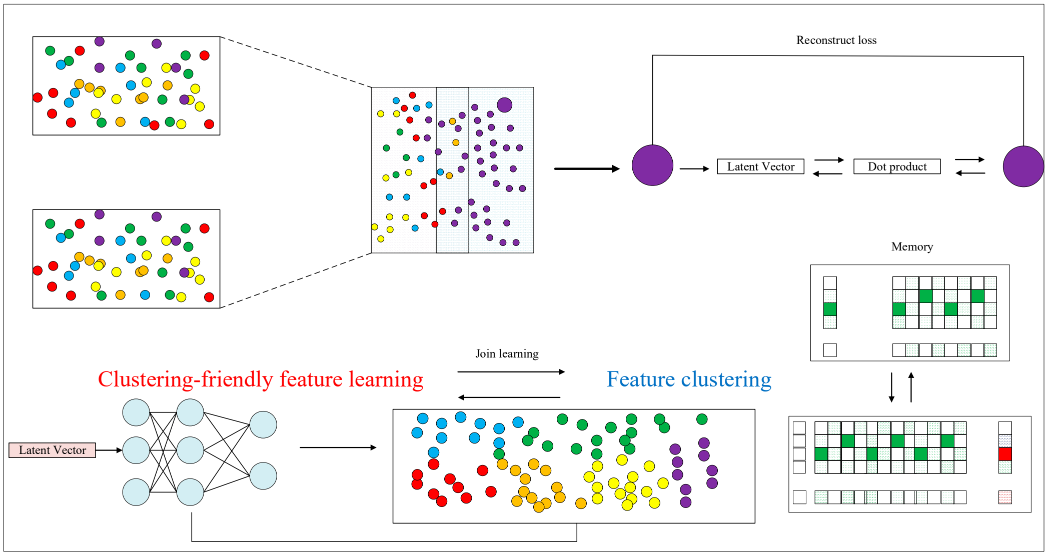

Figure S8 The structure diagram of Deep Clustering algorithm.

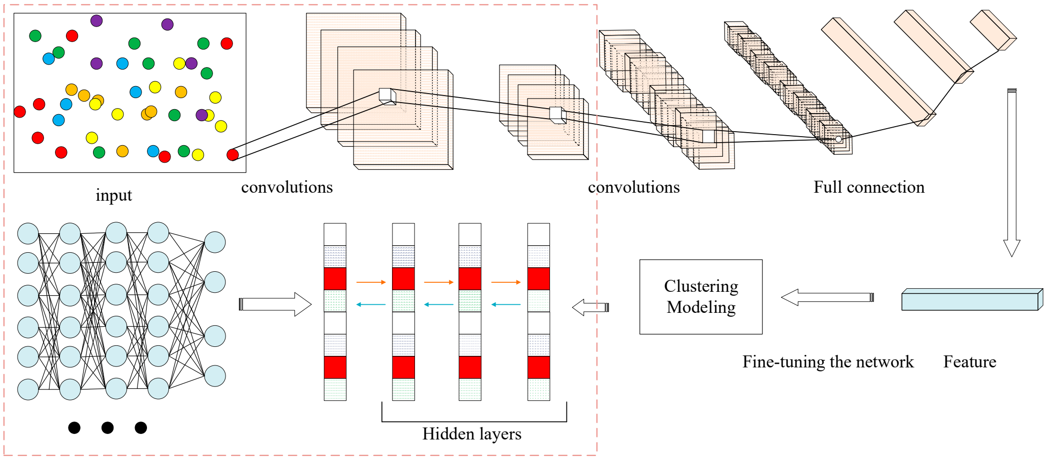

Figure S9 The structure diagram of Deep Clustering Network algorithm.

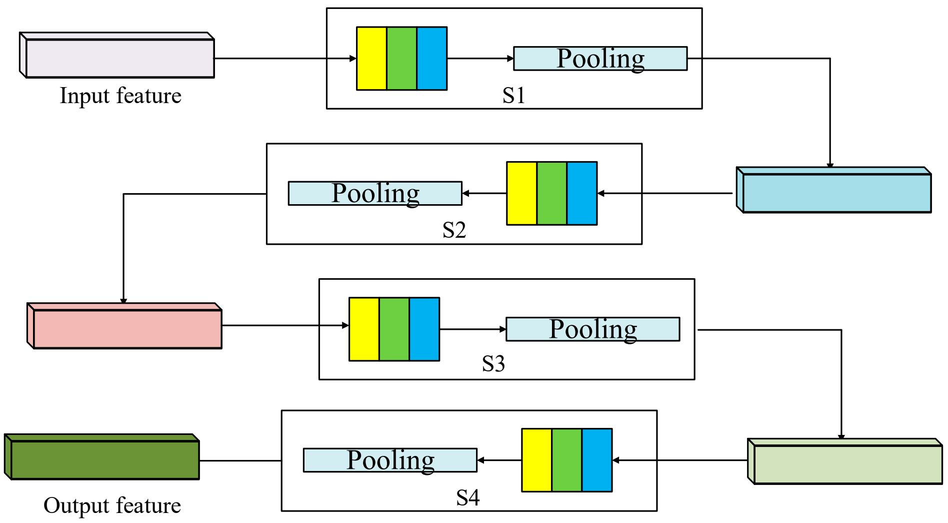

Figure S10 The structure diagram of Joint Unsupervised Learning of Deep Representations and Image Clusters algorithm.
